# Mobilising marine biodiversity data: a new malacological dataset of Italian records (Mollusca)

**DOI:** 10.3897/BDJ.12.e136243

**Published:** 2025-02-28

**Authors:** Arianna Giannini, Massimo Appolloni, Luigi Romani, Marco Oliverio

**Affiliations:** 1 Department of Biology and Biotechnologies "Charles Darwin", Sapienza University, Rome, Italy Department of Biology and Biotechnologies "Charles Darwin", Sapienza University Rome Italy; 2 Civic Museum of Zoology, Rome, Italy Civic Museum of Zoology Rome Italy; 3 Institute of Systematics, Evolution, Biodiversity, Muséum national d'Histoire naturelle, Paris, France Institute of Systematics, Evolution, Biodiversity, Muséum national d'Histoire naturelle Paris France; 4 Via delle Ville, 79 - 55012 Capannori, Lucca, Italy Via delle Ville, 79 - 55012 Capannori Lucca Italy

**Keywords:** marine, mollusca, big data, biodiversity conservation, Natural History Collections, occurrences, Italy

## Abstract

**Background:**

The location and palaeoceanographic history of the Mediterranean Sea make it a biodiversity hotspot, prompting extensive studies in this region. However, despite the marine biodiversity of this area being apparently widely studied, a large amount of distributional data for Mediterranean taxa is still unpublished or scattered in various sources and formats, causing severe limitations to their potential reuse. This emerges as a particularly thorny issue for highly biodiverse and neglected taxa, such as invertebrates. The mobilisation of these frozen data through a process of standardisation and georeferencing could potentially support biodiversity research and conservation. The aim of this work is to provide a standardised pipeline to integrate these dispersed data, focusing on the Italian waters of the Mediterranean Sea and using molluscs as target taxa. Data were gathered from two main sources: published literature and Natural History Collections. The harmonisation process involved three key steps: 1) terminology and structure standardisation; 2) taxonomy updating and 3) georeferencing. Our efforts yielded over 44000 standardised records of mollusc species from Italian seawaters. These records encompassed primary biodiversity data from newly-digitised specimens owned by 11 different institutions and private collectors, as well as secondary biodiversity data extracted from 311 published studies.

**New information:**

This work is the first attempt to mobilise the available distributional information of Italian marine mollusc species from Natural History Collections and literature, converting the retrieved data into point-occurrence records through standard protocols, thus creating a FAIR (Findable, Accessible, Interoperable and Reusable) dataset collating these records from Italian marine sectors.

## Introduction

Human impact on natural ecosystems is leading to several changes in global biodiversity structure and distribution. These changes include the loss of a portion of species large enough to suggest a sixth mass extinction, especially when coupled with the rate at which these extinctions are occurring (Cowie et al. 2022). This situation underscores the urgent need to enhance the quality and quantity of knowledge about species distribution over space and time, which is essential for making sustainable management decisions at various scales, from global to regional. In addition to primary biodiversity data gathered directly from fieldwork, another valuable source for enhancing our understanding of species distribution comes from already published data. However, this information is often published in formats that hinder easy reuse, such as species lists, non-standardised tables or occurrences reported only textually in the article body. The mobilisation of this information in formats that can be integrated and reused produces the so-called secondary biodiversity data (Marques et al. 2024). The possibility to integrate biodiversity data from various sources and formats (e.g. Natural History Collections (NHCs), literature, research and citizen-science programmes) can link the multiple domains of which biodiversity science is composed, such as taxonomy, ecology, conservation and socio-economic realms, filling gaps and offering new perspectives and opportunities (Heberling et al. 2021). With the 21^th^ century, various organisations and facilities promoting the collection and integration of opportunistic biodiversity data have been created (e.g. Edwards et al. (2000)) and, to date, an increasing number of research and conservation programmes rely on point-occurrence records stored in this type of repositories. Although we are in the middle of the big data revolution (Runting et al. 2020), collation efforts remain uneven across geographical regions and taxa. Occurrences are biased towards regions and groups with more resources and a longer tradition of natural history studies (García-Roselló et al. 2023), as well as more accessible areas, resulting in marine systems being less explored (Luypaert et al. 2019). Societal preferences play a crucial role in selecting the taxonomic groups on which to focus funding for data collection (Troudet et al. 2017) and public’s *willingness to pay* is often centred on charismatic taxa, while invertebrates are some of the most neglected ones (Ressurreição et al. 2012), despite being amongst the most diverse and abundant groups. Those factors (with marine invertebrates data remaining frozen in their repositories) are currently impeding an unbiased prioritisation of both global and regional conservation strategies. In this context, in Italy, marine molluscs are often amongst the groups least considered in the development and management of conservation strategies, with the Habitats Directive (1992/43/EEC) deeming only 0.2% of Italian species worthy of conservation and a further 0.6% of species protected solely by the Bern and Barcelona Conventions. In addition to this, only 2% of the Mediterranean malacofauna is assessed according to Red List criteria and, of this percentage, 43% of the species are still Data Deficient (IUCN 2024). Fifteen of the 17 Mediterranean species of conservation concern according to international agrreements were recently assessed according to the IUCN Red List Assessment criterion A3C (population reduction projected to be met in the future based on a decline in area of occupancy) (Schultz et al. 2023), but there is still a long way to go to gain an overall knowledge of the conservation status of the Italian marine malacofauna. As early as 2000, Scotti and Chemello (2000) attempted to raise conservationists' interest, noting that, at that time, no Italian marine mollusc species was assessed in the IUCN Red List, partly attributing this to the little international recognition given to Mediterranean researchers in this period. Today, 24 years later, there is still a lack of information on the conservation status of most of the Italian malacofauna and, consequently, a lack of adequate conservation measures. In this context, the present work proposes to start filling this knowledge gap by building a FAIR dataset by integrating distributional information of marine mollusc species reported in Italy and making them usable as point-occurrence records, from newly-digitised specimens preserved in both public and private Natural History Collections, as well as mobilising secondary biodiversity data from published studies.

## General description

### Purpose

**Figure 1.**
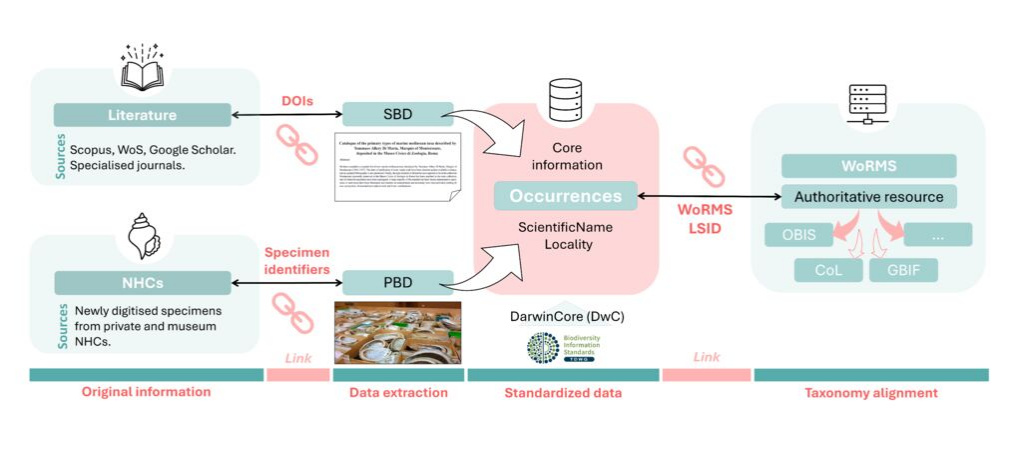
Dataset concept. The dataset's core information consists of species occurrences. Occurrences are extracted from two sources: literature and NHCs. From the original source, records are converted into Primary or Secondary Biodiversity Data (respectively, PBD and SBD) through a process of standardisation and, when necessary, georeferencing. The scientific names of species are aligned with the World Register of Marine Species (WoRMS) nomenclature so that they are comparable with other taxonomic database resources (e.g. Ocean Biodiversity Information System: www.obis.org, Catalogue of Life: www.catalogueoflife.org, Global Biodiversity Information Facility: www.gbif.org).

The present work aims at collecting and making usable in the form of point-occurrences the distributional data of marine mollusc species reported in Italy, by integrating via harmonisation and georeferencing processes both primary (i.e. newly-digitised specimen from public and private Natural History Collections) and secondary biodiversity data (i.e. non-databased spatial information of species reported in publicly-accessible papers). The dataset concept can be visualised in Fig. 1.

## Sampling methods

### Sampling description

Data were gathered from two main sources: literature and Natural History Collections (NHCs). To collect literature data, a comprehensive search was performed on the public databases Scopus and Web of Science. In addition to this, we also searched data from journals specialised on Mediterranean marine fauna, namely *Iberus* and all the volumes of both journals of the Italian Society of Malacology (*Società Italiana di Malacologia*, SIM): *Bollettino Malacologico* and *Alleryana*. Since until the publication of the *Checklist of the Italian Fauna* (Bedulli et al. 1995a, Bedulli et al. 1995b, Bedulli et al. 1995c, Bello 1995, Bodon et al. 1995, Manganelli et al. 1995), no unified standard existed for Italian molluscan taxonomy and nomenclature - and verifying the accuracy of identifications reported in literature would have been difficult without direct check of the actual specimens - the literature search was restricted to publications issued after the first edition of the *Checklist of the Italian Fauna*. Species distribution information published in various formats (e.g. data tables in supplementary materials or within the paper, species lists, statements reporting the species occurrence) were considered as potential raw data. Papers with data already published in public databases were excluded. In order to avoid collecting the same record several times, only papers with new data were considered (i.e. new data derived from a dedicated sampling or non-new data published for the first time). In Table 1, search information are summarised. The full list of papers from which data were gathered is provided in Suppl. material 1. Data from NHCs were collected by direct request to private collectors (Giannini and Oliverio 2022) and institutions. The number of raw records initially received from each NHC owner and the final number of standardised records are listed in Suppl. material 2, together with the code used to identify the institution - derived from the Global Registry of Scientific Collections (Liu et al. 2024) - or private collector in the dataset. From both sources, records were included in the dataset if at least the occurrence locality and a taxonomic identification at the genus level (or more specific) were stated.

### Quality control

To remove human-readable leading, trailing, double spaces and non-printable characters, the entire dataset was run through the Excel TRIM, CLEAN and SUBSTITUTE functions. Carriage returns were checked and removed using Notepad++ software (Ho 2011). To define the consistency and quality of the collected occurrences, a series of cleaning filters and manual checks were applied, which are described in detail in the next section (i.e. Step Description). To establish the quality of the taxonomic data, the resulting list of species in the dataset was extracted and compared with the *Checklist of Italian Fauna* (Renda et al. 2022). The species present in the dataset, but not included in the latter, were checked by expert taxonomists (under the supervision of Marco Oliverio and Luigi Romani), who solved problems and helped remove dubious records. The records underwent two geographic quality checks: the first one pre-georeferencing (Step description section, second step) - performed using the point-radius method (Wieczorek et al. 2010) - in which all records with too vague textual location were removed; and the second one post-georeferencing, in which all records with an uncertainty radius > 5000 m were removed (Step description section, seventh step). The double spatial filtering was necessary as high levels of uncertainty in georeferencing can create problems for spatial analyses (Reside et al. 2011). On the other hand, removing data with high spatial uncertainty can excessively reduce the sample size and, thus, the accuracy of the analyses (Smith et al. 2023) for which the data can be used. The uncertainty limit of 5000 m was chosen as a compromise between the completeness of the dataset and the quality and usability of the data. Some records from private NHCs refer to shells collected by fishermen and it was decided to keep them in the dataset, only if the fishing area/location was recorded and the geographical reliability of such records was acceptable, specifying that the collector is a fisherman in the *dwc:recordedBy* column to ensure transparency. In these cases, raw records are frequently accompanied by two locations: the port of arrival of the fishing vessel or the place where the collector acquired the specimen and the recorded collection area. If only the location of acquisition (and not the actual collection area) was explicitly indicated, the record was excluded from the dataset. This approach was consistent with the treatment applied to specimens obtained through purchase or as gifts, if only the acquisition location was provided. Like all other records, locations that were too vague were excluded during the double geographical quality check.

### Step description

**Figure 2.**
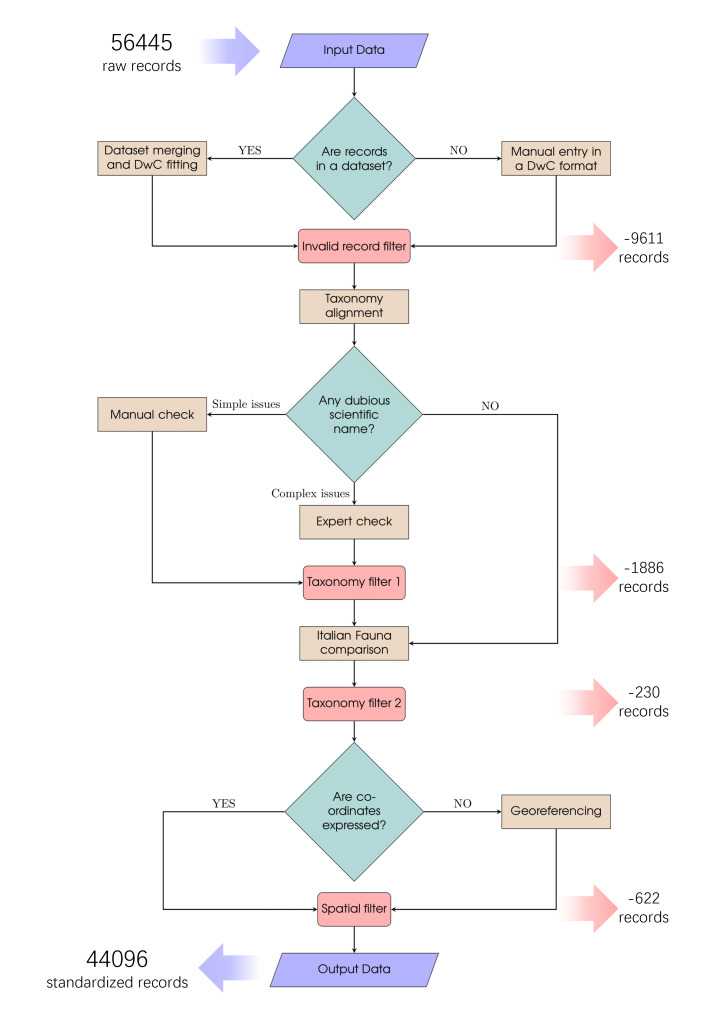
Flow chart of the standardisation pipeline, with the number of input records and the number of discarded ones at each step. With the term "invalid", we identify all records out of scope or with problems (i.e. duplicates, records lacking essential information, fossils, non-marine species, specimens with a higher level of identification than the genus, ambiguous and/or unclear identification, records with dubious locality, occurrences outside study area or with broad locality).

1. Firstly, data were merged and formatted in a Darwin Core scheme (Darwin Core Task Group 2009), using the Biodiversity Data Cleaning toolkit package (Ribeiro et al. 2022) in R (R Core Team 2022, ver. 4.2.2).

2. With the same package, a first filter was performed to clean the dataset from duplicates and records lacking essential information (i.e. identification or locality/coordinates). Then, data were manually filtered to retrieve records that were: out of scope (i.e. occurrences outside the Italian Marine Exclusive Economic Zone, fossils, non-marine species), too vague (i.e. broad locality, specimens with a higher level of identification than the genus) or dubious (dubious locality, ambiguous and/or unclear identification). All these cleaning steps have been consolidated into the "Invalid Records Filter" block in Fig. 2.

3. Taxonomy was aligned to the one proposed by the World Register of Marine Species (WoRMS Editorial Board 2024) using the taxon-match Life Watch webservice (Fuentes and Fiore 2014), also extracting the WoRMS Life Science Identifiers (Vandepitte et al. 2015) for each valid scientific name to trace as far as possible rehashes of taxonomy, which in marine molluscs are quite common, especially through molecular evidence (e.g. Chiappa et al. (2022), Furfaro et al. (2022), Nocella et al. (2024)).

4. The remaining dubious taxonomy that was not automatically validated was checked manually and then submitted to experts, which resulted in the removal of other records with dubious identification.

5. Open Nomenclature (ON) qualifiers (Richter 1948, Matthews 1973, Bengtson 1988) were used to set uncertainty and provisional statuses for taxonomic identifications, applied following Sigovini et al. (2016) guidelines for ON terms harmonisation.

6. Subsequently, records were classified in seven different groups based on the type of the geographic information they had, in order to georeference them by the most appropriate method. Georeferencing was performed following the point-radius method (Wieczorek et al. 2010) and the Zermoglio et al. (2020) protocol, using GEOLocate web-based collaborative client (Rios and Bart 2010) and QGIS (QGIS.org 2024, ver. 3.22.13). Each final processed record has associated coordinates expressed in WGS84 decimal degrees and an uncertainty measure in metres. Table 2 details the method and, where applicable, protocol of georeferencing applied to each of the seven original geographic information classes. The GEBCO_2022 global terrain model was used to georeference depth data correctly (GEBCO Bathymetric Compilation Group 2022).

7. During the georeferencing process it was possible to remove other data occurring outside study boundaries. We then excluded records with >5000 m of uncertainty radius.

8. As raw temporal data from NHCs arrived in various formats, this information was handled with the R package lubridate (Grolemund and Wickham 2011) and converted to ISO 8601 format. The temporal information collected spans various degrees of resolution, from the exact date to time ranges between years. We decided to mantain also records without temporal information. No hourly data were collected. Standardision and cleaning pipeline main steps are reported in Fig. 2.

## Geographic coverage

### Description

**Figure 3.**
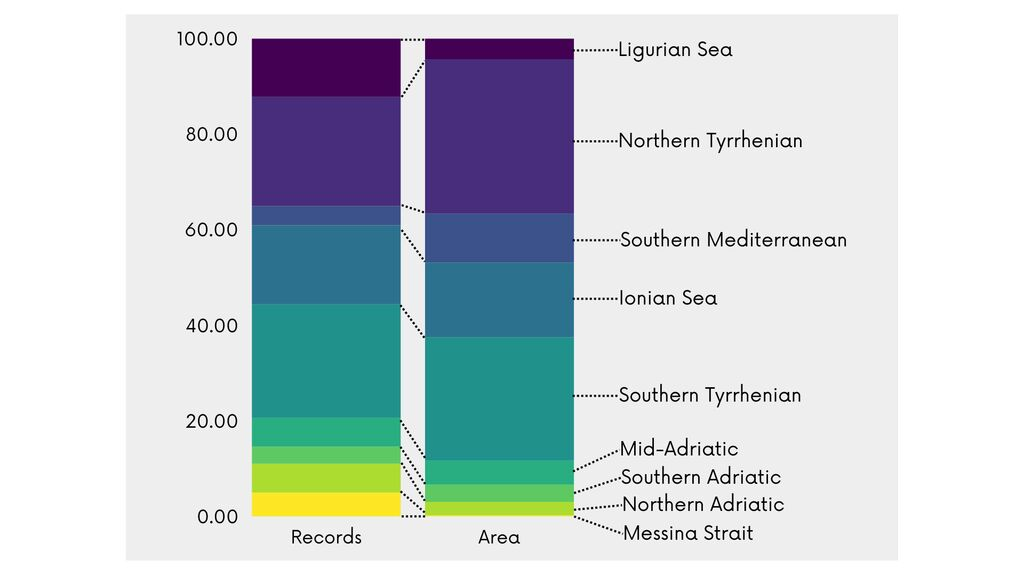
Data abundance (%) per biogeographical sector compared with its extent (%) in the Italian Marine Exclusive Economic Zone.

**Figure 4.**
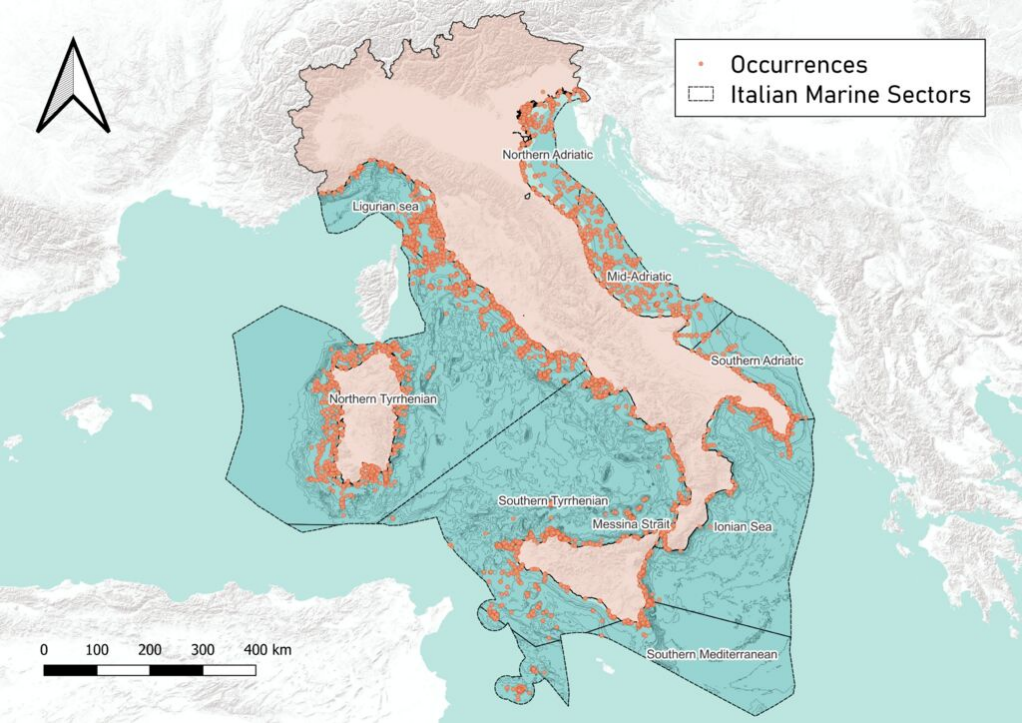
Distribution of collected occurrences.

Collected data occurred within the Italian Exclusive Economic Zone (EEZ), that consists of a marine area of 538,216 km^2^ (Khalfallah et al. 2020) situated in the Mediterranean Sea. This area is divided into nine biogeographical sectors based on the distribution of coastal taxa and the presence of physical, hydrological and physiological barriers (Bianchi 2004, Giacobbe and Oliverio 2024): Ligurian Sea, Northern Tyrrhenian, Southern Tyrrhenian and Messina Strait on the west coast of Italy, Northern Adriatic, Southern Adriatic and Mid-Adriatic on the east coast and Southern Mediterranean and Ionian Sea in the southern part. This subdivision has already been used to study the distribution of both marine plant and animal species in the marine flora and fauna checklist of the Società Italiana di Biologia Marina (Relini 2008, Relini 2010) and for the latest version of the Italian checklist of the marine malacofauna (Renda et al. 2022) and has been proposed as a standard in the Marine Regions Gazetteer (Flanders Marine Institute 2024). Sectors with the highest number of recorded occurrences are the Tyrrhenian Sea (with 10480 occurrences for the Southern area and 10076 for the Northern one), followed by the Ionian Sea (7284), Ligurian Sea (5384), Northern Adriatic (2652), Mid-Adriatic (2647), Messina Strait (2212), Southern Mediterranean (1776) and Southern Adriatic (1585). Since sector areas are very diverse, from the smallest (the Messina Strait) to the largest (the Northern Tyrrhenian Sea) data coverage for each sector may vary depending on its size. Despite this general trend, the Strait of Messina, which covers only 0.2% of the Italian sea surface, contains about 5.0% of the dataset's occurrences, proving to be a particularly well-studied area (Fig. 3). The distribution of collected, cleaned and georeferenced occurrences is shown in Fig. 4.

### Coordinates

35.06440614922465 and 45.80891370810167 Latitude; 5.889722222129848 and 18.99523827942329 Longitude.

## Taxonomic coverage

### Description

The dataset includes 44096 occurrences of 1513 Italian marine mollusc species, covering 85% of the Italian malacofauna and six out of the eight classes reported in Italy (Renda et al. 2022): Gastropoda (1110 species, 32832 occurrences), Bivalvia (326 species, 9410 occurrences), Polyplacophora (28 species, 820 occurrences), Cephalopoda (35 species, 682 occurrences), Scaphopoda (12 species, 331 occurrences) and Monoplacophora (2 species, 21 occurrences) (Table 3). Of the specimens reported in the dataset, 97% are identified at least at species level, while the remaining part is identified at genus level. The complete list of species can be viewed in Suppl. material 3, while Suppl. material 4 contains annotations on the inclusion of some ambiguous taxa (i.e. taxonomic entities that are difficult to interpret/not completely resolved, possible fossil specimens and occasional alien species for the Italian fauna) in the dataset.

### Taxa included

**Table:** 

Rank	Scientific Name	
kingdom	Animalia	
phylum	Mollusca	
class	Bivalvia	
class	Cephalopoda	
class	Gastropoda	
class	Monoplacophora	
class	Polyplacophora	
class	Scaphopoda	
family	Acanthochitonidae	
family	Acteonidae	
family	Addisoniidae	
family	Aegiridae	
family	Aeolidiidae	
family	Aglajidae	
family	Akeridae	
family	Alacuppidae	
family	Amathinidae	
family	Anabathridae	
family	Anatomidae	
family	Anomiidae	
family	Aplysiidae	
family	Aporrhaidae	
family	Architectonicidae	
family	Arcidae	
family	Argonautidae	
family	Arminidae	
family	Assimineidae	
family	Astartidae	
family	Atlantidae	
family	Barleeiidae	
family	Basterotiidae	
family	Bathysciadiidae	
family	Borsoniidae	
family	Brachioteuthidae	
family	Bullidae	
family	Bursidae	
family	Cadlinidae	
family	Caecidae	
family	Calliostomatidae	
family	Callistoplacidae	
family	Callochitonidae	
family	Calmidae	
family	Calycidorididae	
family	Calyptraeidae	
family	Cancellariidae	
family	Capulidae	
family	Cardiidae	
family	Carditidae	
family	Carinariidae	
family	Cassidae	
family	Cavoliniidae	
family	Cerithiidae	
family	Cerithiopsidae	
family	Chamidae	
family	Charoniidae	
family	Chauvetiidae	
family	Chilodontaidae	
family	Chitonidae	
family	Chromodorididae	
family	Cimidae	
family	Cingulopsidae	
family	Clathurellidae	
family	Clavagellidae	
family	Cliidae	
family	Cocculinidae	
family	Colloniidae	
family	Colpodaspididae	
family	Colubrariidae	
family	Columbellidae	
family	Conidae	
family	Corbulidae	
family	Cornirostridae	
family	Coryphellidae	
family	Costellariidae	
family	Cranchiidae	
family	Crassatellidae	
family	Creseidae	
family	Cuspidariidae	
family	Cylichnidae	
family	Cymatiidae	
family	Cymbuliidae	
family	Cypraeidae	
family	Cystiscidae	
family	Dendrodorididae	
family	Dentaliidae	
family	Diaphanidae	
family	Discodorididae	
family	Donacidae	
family	Dorididae	
family	Dotidae	
family	Dreissenidae	
family	Drilliidae	
family	Elachisinidae	
family	Eledonidae	
family	Ellobiidae	
family	Embletoniidae	
family	Entalinidae	
family	Epitoniidae	
family	Eratoidae	
family	Eubranchidae	
family	Eulimidae	
family	Facelinidae	
family	Fasciolariidae	
family	Fionidae	
family	Fissurellidae	
family	Flabellinidae	
family	Fusiturridae	
family	Fustiariidae	
family	Gadilidae	
family	Gadilinidae	
family	Galeommatidae	
family	Gastrochaenidae	
family	Glossidae	
family	Glycymerididae	
family	Goniodorididae	
family	Granulinidae	
family	Gryphaeidae	
family	Haliotidae	
family	Halonymphidae	
family	Haminoeidae	
family	Hancockiidae	
family	Hanleyidae	
family	Heliconoididae	
family	Hermaeidae	
family	Heroidae	
family	Hiatellidae	
family	Histioteuthidae	
family	Horaiclavidae	
family	Hyalocylidae	
family	Hyalogyrinidae	
family	Hydrobiidae	
family	Iravadiidae	
family	Ischnochitonidae	
family	Isognomonidae	
family	Janolidae	
family	Kelliellidae	
family	Laonidae	
family	Larocheidae	
family	Lasaeidae	
family	Lepetellidae	
family	Lepetidae	
family	Leptochitonidae	
family	Limacinidae	
family	Limapontiidae	
family	Limidae	
family	Limopsidae	
family	Littorinidae	
family	Loliginidae	
family	Lottiidae	
family	Lucinidae	
family	Lyonsiellidae	
family	Lyonsiidae	
family	Mactridae	
family	Malleidae	
family	Malletiidae	
family	Mangeliidae	
family	Margaritidae	
family	Marginellidae	
family	Mathildidae	
family	Mesodesmatidae	
family	Mitridae	
family	Mitromorphidae	
family	Murchisonellidae	
family	Muricidae	
family	Myidae	
family	Myrrhinidae	
family	Mytilidae	
family	Nassariidae	
family	Naticidae	
family	Neoleptonidae	
family	Neopilinidae	
family	Neritidae	
family	Newtoniellidae	
family	Noetiidae	
family	Notodiaphanidae	
family	Nuculanidae	
family	Nuculidae	
family	Octopodidae	
family	Octopoteuthidae	
family	Ocythoidae	
family	Omalogyridae	
family	Ommastrephidae	
family	Onchidiidae	
family	Onchidorididae	
family	Onychoteuthidae	
family	Orbitestellidae	
family	Ostreidae	
family	Otinidae	
family	Ovulidae	
family	Oxynoidae	
family	Pandoridae	
family	Parilimyidae	
family	Patellidae	
family	Pectinidae	
family	Pediculariidae	
family	Pendromidae	
family	Peraclidae	
family	Periplomatidae	
family	Pharidae	
family	Phasianellidae	
family	Philinidae	
family	Pholadidae	
family	Phyllidiidae	
family	Pinnidae	
family	Pisaniidae	
family	Piseinotecidae	
family	Plakobranchidae	
family	Planaxidae	
family	Platyhedylidae	
family	Pleurobranchaeidae	
family	Pleurobranchidae	
family	Polyceridae	
family	Poromyidae	
family	Potamididae	
family	Pristiglomidae	
family	Propeamussiidae	
family	Psammobiidae	
family	Pseudococculinidae	
family	Pteriidae	
family	Pterotracheidae	
family	Pulsellidae	
family	Pyramidellidae	
family	Ranellidae	
family	Raphitomidae	
family	Retusidae	
family	Rhizoridae	
family	Ringiculidae	
family	Rissoellidae	
family	Rissoidae	
family	Rissoinidae	
family	Runcinidae	
family	Samlidae	
family	Scaliolidae	
family	Scaphandridae	
family	Scissurellidae	
family	Scyllaeidae	
family	Semelidae	
family	Sepiidae	
family	Sepiolidae	
family	Siliquariidae	
family	Siphonariidae	
family	Skeneidae	
family	Skeneopsidae	
family	Solecurtidae	
family	Solemyidae	
family	Solenidae	
family	Spondylidae	
family	Tellinidae	
family	Teredinidae	
family	Tethydidae	
family	Thraciidae	
family	Thyasiridae	
family	Thysanoteuthidae	
family	Tjaernoeiidae	
family	Tonicellidae	
family	Tonnidae	
family	Tornidae	
family	Trapezidae	
family	Tremoctopodidae	
family	Trimusculidae	
family	Trinchesiidae	
family	Triphoridae	
family	Tritoniidae	
family	Triviidae	
family	Trochaclididae	
family	Trochidae	
family	Truncatellidae	
family	Tudiclidae	
family	Turbinidae	
family	Turritellidae	
family	Tylodinidae	
family	Umbraculidae	
family	Ungulinidae	
family	Vanikoridae	
family	Velutinidae	
family	Veneridae	
family	Vermetidae	
family	Verticordiidae	
family	Vitrinellidae	
family	Volvatellidae	
family	Xenophoridae	
family	Xylodisculidae	
family	Xylophagaidae	
family	Yoldiidae	

## Temporal coverage

**Data range:** 1814-1-01 – 2023-12-31.

### Notes

**Figure 5.**
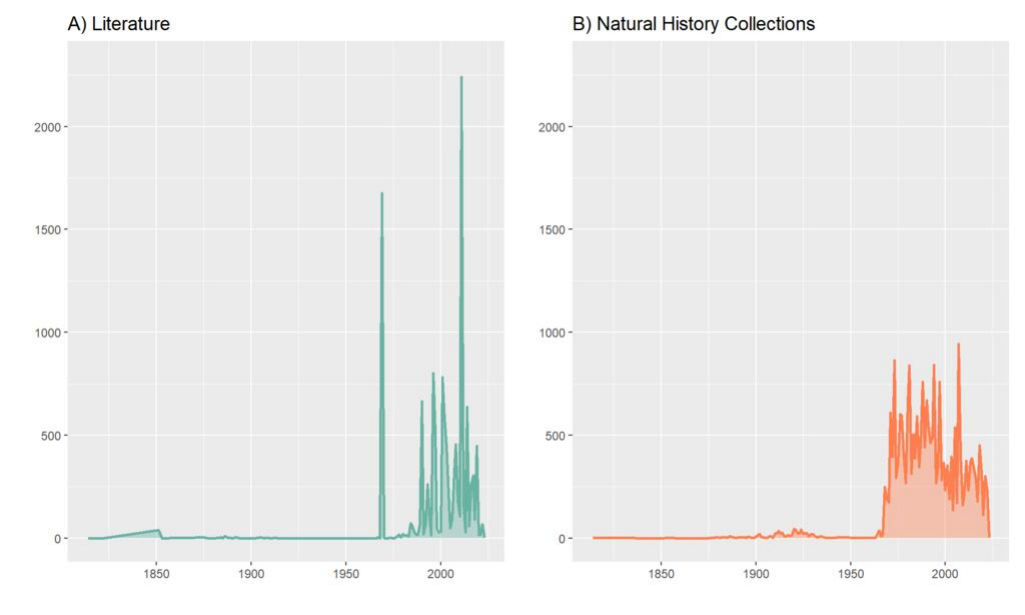
Temporal coverage of the data, divided by data obtained through literature search (A) and from Natural History Collections (B).

The dataset includes both recent and historical data. Regarding NHCs data, pre-1950 records come from the historical collections held in the Civic Museum of Zoology of Rome (i.e. Monterosato, Meli and Piersanti NHCs), while post-1950 records are from private collections. For literature data, on the other hand, pre-1950 records come mainly from published catalogues of historical collections, while more recent records come from faunistic and ecological studies. The list of all literature and NHCs data sources can be found respectively in Suppl. material 1 and in Suppl. material 2. The number of records per year obtained from literature and from NHCs can be visualised in Fig. 5.

## Usage licence

### Usage licence

Other

### IP rights notes

**Creative Commons Attribution Non-Commercial (CC-BY-NC 4.0)**

## Data resources

### Data package title

Mobilising marine biodiversity data: a new malacological dataset of Italian records (Mollusca)

### Resource link

https://www.gbif.org/dataset/e0370da7-b32b-414c-8a61-1d86125075f3

### Alternative identifiers

https://cloud.gbif.org/eca/resource?r=mzur_sap_zoo_01

### Number of data sets

1

### Data set 1.

#### Data set name

Mobilising marine biodiversity data: a new malacological dataset of Italian records (Mollusca)

#### Data format

Darwin Core Archive

**Data set 1.** 

Column label	Column description
institutionID	An identifier for the institution having custody of the object(s) or information referred to in the record. For data collected from public institutions, the identifiers of the Global Registry of Scientific Collections (GRSciColl) were used.
institutionCode	The name (or acronym) in use by the institution having custody of the object(s) or information referred to in the record. For data collected from public institutions, the identifiers of the Global Registry of Scientific Collections (GRSciColl) were used. For data collected from private collections, the following non-standard identifiers were used, marked with the prefix "PriColl_": Nofroni I. (PriColl_NI), Renda W. (PriColl_RW), Romani L. (PriColl_RL), Roncone F. (PriColl_RF), Russo P. (PriColl_RP), Tringali L. (PriColl_TL), Trono D. (PriColl_TD).
collectionCode	The name, acronym, coden or initialism identifying the collection or dataset from which the record was derived.
basisOfRecord	The specific nature of the data record.
catalogNumber	An identifier for the record within the dataset or collection.
recordedBy	A list (concatenated and separated) of names of people, groups or organisations responsible for recording the original dwc:Occurrence.
individualCount	The number of individuals present at the time of the dwc:Occurrence.
establishmentMeans	Statement about whether a dwc:Organism has been introduced to a given place and time through the direct or indirect activity of modern humans.
associatedReferences	A list (concatenated and separated) of identifiers (publication, bibliographic reference, global unique identifier, URI) of literature associated with the dwc:Occurrence. This column was used to indicate the bibliographic source from which literature data was collected.
eventDate	The date or interval during which a dwc:Event occurred. Dates are expressed following the ISO 8601 standard.
year	The four-digit year in which the dwc:Event occurred, according to the Common Era Calendar.
higherGeographyID	The Getty Thesaurus of Geographic Names persistent identifier for the geographic region within which the dcterms:Location occurred.
higherGeography	The less specific geographic name of the information captured in the dwc:locality term.
waterBody	The name of the water body in which the dcterms:Location occurs. Names of the Italian marine biogeographical areas were used (Bianchi et al. 2004).
country	The name of the country in which the dcterms:Location occurs.
stateProvince	The name of the next smaller administrative region than country (i.e. region) in which the dcterms:Location occurs.
locality	The specific description of the place.
minimumDepthInMetres	The lesser depth of a range of depth below the local surface, in metres.
maximumDepthInMetres	The greater depth of a range of depth below the local surface, in metres.
decimalLatitude	The geographic latitude (in decimal degrees, using the spatial reference system given in dwc:geodeticDatum) of the geographic centre of a dcterms:Location.
decimalLongitude	The geographic longitude (in decimal degrees, using the spatial reference system given in dwc:geodeticDatum) of the geographic centre of a dcterms:Location.
geodeticDatum	The ellipsoid, geodetic datum or spatial reference system (SRS), upon which the geographic coordinates given in dwc:decimalLatitude and dwc:decimalLongitude are based.
coordinateUncertaintyInMetres	The horizontal distance (in metres) from the given dwc:decimalLatitude and dwc:decimalLongitude describing the smallest circle containing the whole of the dcterms:Location.
georeferencedBy	A list (concatenated and separated) of names of people, groups or organisations who determined the georeference (spatial representation) for the dcterms:Location.
georeferenceProtocol	A description or reference to the methods used to determine the spatial footprint, coordinates and uncertainties.
georeferenceRemarks	Notes or comments about the spatial description determination, explaining assumptions made in addition or opposition to the those formalised in the method referred to in dwc:georeferenceProtocol.
identificationQualifier	A brief phrase or a standard term ("cf.", "aff.") to express the determiner's doubts about the dwc:Identification. The standard terminology proposed by Sigovini et al. (2016) was followed.
identifiedBy	A list (concatenated and separated) of names of people, groups or organisations who assigned the dwc:Taxon to the subject.
scientificNameID	An identifier for the nomenclatural (not taxonomic) details of a scientific name. WoRMS LSID persistent identifiers were used.
scientificName	The full scientific name.
class	The full scientific name of the class in which the dwc:Taxon is classified.
order	The full scientific name of the order in which the dwc:Taxon is classified.
family	The full scientific name of the family in which the dwc:Taxon is classified.
genus	The full scientific name of the genus in which the dwc:Taxon is classified.
specificEpithet	The name of the first or species epithet of the dwc:scientificName.
infraspecificEpithet	The name of the lowest or terminal infraspecific epithet of the dwc:scientificName.
taxonRank	The taxonomic rank of the most specific name in the dwc:scientificName.
scientificNameAuthorship	The authorship information for the dwc:scientificName formatted according to the conventions of the applicable dwc:nomenclaturalCode.

## Supplementary Material

74CC3EC4-F0F0-5188-AF96-ED11E49E435A 10.3897/BDJ.12.e136243.suppl1 Supplementary material 1 Sources from which literature data were obtained Data type Table Brief description References of the 311 publications from which data were extracted and number of records obtained from each source. File: oo_1189435.xlsx https://binary.pensoft.net/file/1189435 Arianna Giannini

17AD6BD9-E73D-53DA-BBD7-CBE66F3640D6 10.3897/BDJ.12.e136243.suppl2 Supplementary material 2 Sources from which NHCs data were obtained Data type Table Brief description List of private collectors and institutions that own the 11 NHCs from which the data were collected, with initial number of raw records and final number of standardised records. File: oo_1189436.xlsx https://binary.pensoft.net/file/1189436 Arianna Giannini

775D9062-A9B7-5F9E-88F8-55CFED310876 10.3897/BDJ.12.e136243.suppl3 Supplementary material 3 Species list Data type Species list File: oo_1123257.pdf https://binary.pensoft.net/file/1123257 Arianna Giannini, Luigi Romani, Marco Oliverio

2A8163C7-6E51-5E0D-995E-35D2C7D9B81A 10.3897/BDJ.12.e136243.suppl4 Supplementary material 4 Notes on some taxa included in the dataset Data type List Brief description This list contains annotations on some ambiguous taxa (i.e. taxonomic entities that are difficult to interpret/not fully resolved, possible fossil taxa and occasional alien species for the Italian fauna) that have nevertheless been included in the dataset. File: oo_1192697.xlsx https://binary.pensoft.net/file/1192697 Arianna Giannini

## Figures and Tables

**Table 1.** Literature data search constraints, dates and number of results obtained for each consulted source.

Source	Search constraints	Start date	N° of results
Scopus	TITLE-ABS-KEY (marine AND mollusca AND italy)	16/05/2023	306
Web of Science	((ALL=(marine)) AND ALL=(mollusca)) AND ALL=(italy)	05/07/2023	442
Google Scholar	MARINE+MOLLUSCA+ITALY source:*Iberus*, from 1995 to 2023.	20/10/2023	33
SIM	All volumes of *Bollettino Malacologico* and *Alleryana* from 1995 to 2023.	24/11/2023	567
**Total**			**1348**

**Table 2.** Description of the seven types of geographic information contained in the original records and georeferencing protocols and methods applied in each case. This information can be found in the dataset column *dwc:georeferenceRemarks*.

Type	Description	Georeference method and protocol	N° of records
Corrected	The original coordinates placed the raw record on land.	The record was moved to the position at sea nearest to the one defined by the original coordinates. A standard uncertainty of 100 m was assigned to the record.	748
Depth driven	The original geographical information was provided as a textual locality and the depth at which the specimen was found.	The record was georeferenced following the Zermoglio et al. (2020) protocol.	21144
Distance driven	The original geographical information was provided as the distance from a locality.	The record was georeferenced following the Zermoglio et al. (2020) protocol.	262
Exact	The raw record already had exact coordinates provided in some format.	Where necessary, coordinates were converted to WGS84 decimal degrees. A standard uncertainty of 50 m was assigned to the record.	9012
Locality approximation	The original geographical information was provided as a textual locality, without depth or distance from the coast.	The record was georeferenced following the Zermoglio et al. (2020) protocol.	9430
Map approximation	The raw record was geographically positioned through a visual representation (e.g. map, satellite imagery) in the original source.	The record has been mapped trying to recreate as closely as possible the position represented in the original source. A standard uncertainty of 500 m was assigned to the record.	2894
Route	The raw record was geographically defined by the coordinates of the start and end of the route taken during the event in which the specimen was found.	The record was georeferenced following the Zermoglio et al. (2020) protocol.	606

**Table 3.** Number of genera, species, subspecies and occurrences per class.

Class	Genus	Species	Subspecies	Occurrences
Bivalvia	207	326	1	9410
Cephalopoda	28	35	0	682
Gastropoda	457	1110	7	32832
Monoplacophora	2	2	0	21
Polyplacophora	12	28	0	820
Scaphopoda	7	12	0	331
**Total**	**713**	**1513**	**8**	**44096**
