# Supplementary material for: Mobilising marine biodiversity data: a new malacological dataset of Italian records (Mollusca)
Source: Biodivers Data J. 2025 Feb 28;13:e136243. doi: 10.3897/BDJ.12.e136243 (PMC11889433; doi:10.3897/BDJ.12.e136243)
Supplement: Supplementary material 3 — Species list [file bdj-13-e136243-s003.pdf]

Class **Polyplacophora**

Order **Callochitonida**

Family **Callochitonidae**

Callochiton calcatus Dell'Angelo & Palazzi, 1994

Callochiton doriae (Capellini, 1859)

Callochiton stefaniae Dell'Angelo, Renda, Sosso, Sigwart & Giacobbe, 2017

Order **Chitonida**

Family **Acanthochitonidae**

Acanthochitona crinita (Pennant, 1777)

Acanthochitona fascicularis (Linnaeus, 1767)

Acanthochitona oblonga Leloup, 1981

Acanthochitona pilosa Schmidt-Petersen, Schwabe & Haszprunar, 2015

Family **Callistoplacidae**

Callistochiton pachylasmae (Monterosato, 1879)

Family **Chitonidae**

Rhyssoplax corallina (Risso, 1826)

Rhyssoplax olivacea (Spengler, 1797)

Rhyssoplax phaseolina (Monterosato, 1879)

Family **Ischnochitonidae**

Connexochiton platynomenus Kaas, 1979

Ischnochiton rissoi (Payraudeau, 1826)

Ischnochiton usticensis Dell'Angelo & Castriota, 1999

Stenosemus dolii (Van Belle & Dell'Angelo, 1998)

Family **Tonicellidae**

Lepidochitona caprearum (Scacchi, 1836)

Lepidochitona cinerea (Linnaeus, 1767)

Lepidochitona furtiva (Monterosato, 1879)

Lepidochitona monterosatoi Kaas & Van Belle, 1981

Order **Lepidopleurida**

Family **Hanleyidae**

Hanleya hanleyi (W. Bean, 1844)

Hanleya mediterranea Sirenko, 2014

Family **Leptochitonidae**

Lepidopleurus cajetanus (Poli, 1791)

Leptochiton algesirensis (Capellini, 1859)

Leptochiton cancellatus (G. B. Sowerby II, 1840)

Leptochiton cimicoides (Monterosato, 1879)

Leptochiton geronensis Kaas & Van Belle, 1985

Leptochiton scabridus (Jeffreys, 1880)

Parachiton africanus (Nierstrasz, 1906)

Class **Monoplacophora**

Order **Neopilinida**

Family **Neopilinidae**

Micropilina minuta Warén, 1989

Veleropilina reticulata (G. Seguenza, 1876)

Class **Cephalopoda**

Superorder **Decapodiformes**

Order **Myopsida**

Family **Loliginidae**

Alloteuthis media (Linnaeus, 1758)

Loligo forbesii Steenstrup, 1856

Loligo vulgaris Lamarck, 1798

Order **Oegopsida**

Family **Brachioteuthidae**

Brachioteuthis riisei (Steenstrup, 1882)

Family **Cranchiidae**

Galiteuthis armata Joubin, 1898

Family **Histioteuthidae**

Histioteuthis bonnellii (A. Férussac, 1835)

Histioteuthis reversa (A. E. Verrill, 1880)

Family **Octopoteuthidae**

Octopoteuthis sicula Rüppell, 1844

Family **Ommastrephidae**

Illex coindetii (Vérany, 1839)

Todarodes sagittatus (Lamarck, 1798)

Todaropsis eblanae (Ball, 1841)

Family **Onychoteuthidae**

Ancistroteuthis lichtensteinii (A. Férussac [in A. Férussac & d'Orbigny], 1835)

Family **Thysanoteuthidae**

Thysanoteuthis rhombus Troschel, 1857

Order **Sepiida**

Family **Sepiidae**

Rhombosepion elegans (Blainville, 1827)

Rhombosepion orbignyanum (A. Férussac, 1826)

Sepia officinalis Linnaeus, 1758

Family **Sepiolidae**

Heteroteuthis dispar (Rüppell, 1844)

Neorossia caroli (Joubin, 1902)

Rondeletiola minor (Naef, 1912)

Rossia macrosoma (Delle Chiaje, 1830)

Sepietta neglecta Naef, 1916

Sepietta obscura Naef, 1916

Sepietta oweniana (A. d'Orbigny, 1841)

Sepiola rondeletii Leach, 1817

Superorder **Octopodiformes**

Order **Octopoda**

Family **Argonautidae**

Argonauta argo Linnaeus, 1758

Family **Eledonidae**

Eledone cirrhosa (Lamarck, 1798)

Eledone moschata (Lamarck, 1798)

Family **Octopodidae**

Callistoctopus macropus (Risso, 1826)

Macrotritopus defilippi (Vérany, 1851)

Octopus salutii Vérany, 1839

Octopus vulgaris Cuvier, 1797

Pteroctopus tetracirrhus (Delle Chiaje, 1830)

Scaeurgus unicirrhus (Delle Chiaje, 1841)

Family **Ocythoidae**

Ocythoe tuberculata Rafinesque, 1814

Family **Tremoctopodidae**

Tremoctopus gracilis (Souleyet, 1852)

Class **Bivalvia**

Subclass **Protobranchia**

Order **Nuculanida**

Family **Malletiidae**

Katadesmia cuneata (Jeffreys, 1876)

Malletia pianii (van Aartsen & Giannuzzi-Savelli, 1991)

Family **Nuculanidae**

Ledella marisnostri La Perna, 2004

Ledella messanensis (Jeffreys, 1870)

Lembulus pella (Linnaeus, 1758)

Saccella commutata (R. A. Philippi, 1844)

Family **Pristiglomidae**

Pristigloma minima (G. Seguenza, 1877)

Family **Yoldiidae**

Microgloma guilonardi (D. F. Hoeksema, 1993)

Microgloma pusilla (Jeffreys, 1879)

Yoldiella frigida (Torell, 1859)

Yoldiella lucida (Lovén, 1846)

Yoldiella nana (M. Sars, 1865)

Yoldiella ovulum La Perna, 2004

Yoldiella philippiana (Nyst, 1845)

Yoldiella striolata (Brugnone, 1876)

Yoldiella wareni La Perna, 2004

Order **Nuculida**

Family **Nuculidae**

Austronucula perminima (Monterosato, 1875)

Ennucula aegeensis (Forbes, 1844)

Ennucula corbuloides (G. Seguenza, 1877)

Nucula hanleyi Winckworth, 1931

Nucula nitidosa Winckworth, 1930

Nucula nucleus (Linnaeus, 1758)

Nucula sulcata Bronn, 1831

Order **Solemyida**

Family **Solemyidae**

Solemya togata (Poli, 1791)

Subclass **Autobranchia**

Infraclass **Pteriomorphia**

Order **Arcida**

Family **Arcidae**

Acar clathrata (Defrance, 1816)

Anadara corbuloides (Monterosato, 1881)

Anadara gibbosa (Reeve, 1844)

Anadara kagoshimensis (Tokunaga, 1906)

Anadara transversa (Say, 1822)

Arca noae Linnaeus, 1758

Asperarca magdalenae La Perna, 1998

Asperarca nodulosa (O. F. Müller, 1776)

Asperarca secreta La Perna, 1998

Barbatia barbata (Linnaeus, 1758)

Bathyarca pectunculoides (Scacchi, 1835)

Bathyarca philippiana (Nyst, 1848)

Tetrarca tetragona (Poli, 1795)

Family **Glycymerididae**

Glycymeris bimaculata (Poli, 1795)

Glycymeris glycymeris (Linnaeus, 1758)

Glycymeris nummaria (Linnaeus, 1758)

Glycymeris pilosa (Linnaeus, 1767)

Family **Limopsidae**

Limopsis aurita (Brocchi, 1814)

Limopsis minuta (R. A. Philippi, 1836)

Family **Noetiidae**

Striarca lactea (Linnaeus, 1758)

Order **Limida**

Family **Limidae**

Acesta excavata (J. C. Fabricius, 1779)

Lima lima (Linnaeus, 1758)

Limaria hians (Gmelin, 1791)

Limaria loscombi (G. B. Sowerby I, 1823)

Limaria tuberculata (Olivi, 1792)

Limatula gwyni (Sykes, 1903)

Limatula subauriculata (Montagu, 1808)

Limatula subovata (Monterosato, 1875)

Limea clandestina (C. Salas, 1994)

Limea crassa (Forbes, 1844)

Order **Mytilida**

Family **Mytilidae**

Amygdalum politum (A. E. Verrill & S. Smith, 1880)

Arcuatula senhousia (W. H. Benson, 1842)

Brachidontes pharaonis (P. Fischer, 1870)

Crenella arenaria Monterosato, 1875

Crenella pellucida (Jeffreys, 1859)

Dacrydium hyalinum (Monterosato, 1875)

Gregariella petagnae (Scacchi, 1832)

Gregariella semigranata (Reeve, 1858)

Idas cristiani Fr. Giusti, Mietto & Sbrana, 2012

Idas cylindricus Pelorce & Poutiers, 2009

Idas emmae Fr. Giusti, Mietto & Sbrana, 2012

Idas filippoi Fr. Giusti, Mietto & Sbrana, 2012

Idas ghisottii Warén & Carrozza, 1990

Idas jaclinae Fr. Giusti, Mietto & Sbrana, 2012

Idas modiolaeformis (Sturany, 1896)

Leiosolenus aristatus (Dillwyn, 1817)

Lioberus agglutinans (Cantraine, 1835)

Lithophaga lithophaga (Linnaeus, 1758)

Modiolula phaseolina (R. A. Philippi, 1844)

Modiolus adriaticus Lamarck, 1819

Modiolus barbatus (Linnaeus, 1758)

Musculus costulatus (Risso, 1826)

Musculus subpictus (Cantraine, 1835)

Mytilaster lineatus (Gmelin, 1791)

Mytilaster marioni (Locard, 1889)

Mytilaster minimus (Poli, 1795)

Mytilaster solidus Monterosato, 1883

Mytilus galloprovincialis Lamarck, 1819

Nypamodiolus simpsoni (J. T. Marshall, 1900)

Perna perna (Linnaeus, 1758)

Rhomboidella prideauxi (Leach, 1815)

Xenostrobus securis (Lamarck, 1819)

Order **Ostreida**

Family **Gryphaeidae**

Neopycnodonte cochlear (Poli, 1795)

Family **Isognomonidae**

Isognomon bicolor (C. B. Adams, 1845)

Family **Malleidae**

Malleus sp.

Family **Margaritidae**

Pinctada fucata (A. Gould, 1850)

Pinctada imbricata Röding, 1798

Pinctada margaritifera (Linnaeus, 1758)

Pinctada radiata (Leach, 1814)

Family **Ostreidae**

Crassostrea virginica (Gmelin, 1791)

Magallana gigas (Thunberg, 1793)

Ostrea edulis Linnaeus, 1758

Ostrea stentina Payraudeau, 1826

Family **Pinnidae**

Atrina fragilis (Pennant, 1777)

Pinna nobilis Linnaeus, 1758

Pinna rudis Linnaeus, 1758

Family **Pteriidae**

Pteria hirundo (Linnaeus, 1758)

Order **Pectinida**

Family **Anomiidae**

Anomia ephippium Linnaeus, 1758

Heteranomia squamula (Linnaeus, 1758)

Pododesmus patelliformis (Linnaeus, 1761)

Family **Pectinidae**

Aequipecten commutatus (Monterosato, 1875)

Aequipecten opercularis (Linnaeus, 1758)

Delectopecten vitreus (Gmelin, 1791)

Flexopecten flexuosus (Poli, 1795)

Flexopecten glaber (Linnaeus, 1758)

Flexopecten hyalinus (Poli, 1795)

Karnekampia sulcata (O. F. Müller, 1776)

Manupecten pesfelis (Linnaeus, 1758)

Mimachlamys varia (Linnaeus, 1758)

Palliolum incomparabile (Risso, 1826)

Palliolum striatum (O. F. Müller, 1776)

Pecten jacobaeus (Linnaeus, 1758)

Pseudamussium clavatum (Poli, 1795)

Pseudamussium peslutrae (Linnaeus, 1771)

Talochlamys multistriata (Poli, 1795)

amily **Propeamussiidae**

Cyclopecten brundisiensis Smriglio & Mariottini, 1990

Cyclopecten hoskynsi (Forbes, 1844)

Parvamussium fenestratum (Forbes, 1844)

Similipecten similis (Laskey, 1811)

Family **Spondylidae**

Spondylus gaederopus Linnaeus, 1758

Spondylus gussonii O. G. Costa, 1830

Subclass **Autobranchia**

Superorder **Imparidentia**

Order **Adapedonta**

Family **Hiatellidae**

Hiatella arctica (Linnaeus, 1767)

Hiatella rugosa (Linnaeus, 1767)

Panopea glycimeris (Born, 1778)

Family **Pharidae**

Ensis ensis (Linnaeus, 1758)

Ensis minor (Chenu, 1843)

Pharus legumen (Linnaeus, 1758)

Phaxas pellucidus (Pennant, 1777)

Family **Solenidae**

Solen marginatus Pulteney, 1799

Order **Cardiida**

Family **Cardiidae**

Acanthocardia aculeata (Linnaeus, 1758)

Acanthocardia deshayesii (Payraudeau, 1826)

Acanthocardia echinata (Linnaeus, 1758)

Acanthocardia paucicostata (G. B. Sowerby II, 1834)

Acanthocardia spinosa ([Lightfoot], 1786)

Acanthocardia tuberculata (Linnaeus, 1758)

Cerastoderma glaucum (Bruguière, 1789)

Fulvia fragilis (Forsskål, 1775)

Laevicardium oblongum (Gmelin, 1791)

Papillicardium minimum (R. A. Philippi, 1836)

Papillicardium papillosum (Poli, 1791)

Parvicardium carrozzai van Aartsen & Goud, 2001

Parvicardium exiguum (Gmelin, 1791)

Parvicardium pinnulatum (Conrad, 1831)

Parvicardium scabrum (R. A. Philippi, 1844)

Parvicardium scriptum (Bucquoy, Dautzenberg & Dollfus, 1892)

Family **Donacidae**

Donax semistriatus Poli, 1795

Donax trunculus Linnaeus, 1758

Donax variegatus (Gmelin, 1791)

Donax venustus Poli, 1795

Family **Psammobiidae**

Gari costulata (W. Turton, 1822)

Gari depressa (Pennant, 1777)

Gari fervensis (Gmelin, 1791)

Gari tellinella (Lamarck, 1818)

Family **Semelidae**

Abra alba (W. Wood, 1802)

Abra longicallus (Scacchi, 1835)

Abra nitida (O. F. Müller, 1776)

Abra prismatica (Montagu, 1808)

Abra segmentum (Récluz, 1843)

Abra tenuis (Montagu, 1803)

Ervilia castanea (Montagu, 1803)

Scrobicularia cottardii (Payraudeau, 1826)

Scrobicularia plana (da Costa, 1778)

Theora lubrica A. Gould, 1861

Family **Solecurtidae**

Azorinus chamasolen (da Costa, 1778)

Solecurtus candidus (Brocchi, 1814)

Solecurtus scopula (W. Turton, 1822)

Solecurtus strigilatus (Linnaeus, 1758)

Family **Tellinidae**

Arcopagia crassa (Pennant, 1777)

Arcopella balaustina (Linnaeus, 1758)

Asbjornsenia pygmaea (Lovén, 1846)

Bosemprella incarnata (Linnaeus, 1758)

Fabulina fabula (Gmelin, 1791)

Gastrana fragilis (Linnaeus, 1758)

Macomangulus tenuis (da Costa, 1778)

Macomopsis cumana (O. G. Costa, 1830)

Moerella distorta (Poli, 1791)

Moerella donacina (Linnaeus, 1758)

Moerella pulchella (Lamarck, 1818)

Peronaea planata (Linnaeus, 1758)

Peronidia albicans (Gmelin, 1791)

Serratina serrata (Brocchi, 1814)

Order **Galeommatida**

Family **Basterotiidae**

Saxicavella jeffreysi Winckworth, 1930

Family **Galeommatidae**

Galeomma turtoni W. Turton, 1825

Family **Lasaeidae**

Arculus sykesii (Chaster, 1895)

Bornia geoffroyi (Payraudeau, 1826)

Bornia sebetia (O. G. Costa, 1830)

Coracuta obliquata (Chaster, 1897)

Draculamya carlosbranai Siragusa & Micali, 2021

Epilepton clarkiae (W. Clark, 1852)

Epilepton parrussetense Giribet & Peñas, 1999

Epilepton subtrigonum (P. Fischer, 1874)

Hemilepton nitidum (W. Turton, 1822)

Kellia suborbicularis (Montagu, 1803)

Kelliopsis jozinae van Aartsen & Carrozza, 1997

Kurtiella bidentata (Montagu, 1803)

Kurtiella pellucida (Jeffreys, 1881)

Kurtiella tumidula (Jeffreys, 1866)

Lasaea adansoni (Gmelin, 1791)

Lepton squamosum (Montagu, 1803)

Litigiella glabra (P. Fischer, 1873)

Mioerycina coarctata (S. V. Wood, 1851)

Montacuta goudi van Aartsen, 1997

Montacuta substriata (Montagu, 1808)

Scacchia oblonga (R. A. Philippi, 1836)

Tellimya ferruginosa (Montagu, 1808)

Tellimya semirubra (Gaglini, 1992)

Order **Gastrochaenida**

Family **Gastrochaenidae**

Rocellaria dubia (Pennant, 1777)

Order **Lucinida**

Family **Lucinidae**

Ctena decussata (O. G. Costa, 1829)

Loripes orbiculatus Poli, 1795

Loripinus fragilis (R. A. Philippi, 1836)

Lucinella divaricata (Linnaeus, 1758)

Lucinoma borealis (Linnaeus, 1767)

Lucinoma spelaeum Palazzi & Villari, 2001

Megaxinus unguiculinus Pallary, 1904

Myrtea spinifera (Montagu, 1803)

Family **Thyasiridae**

Axinulus alleni (Carrozza, 1981)

Axinulus croulinensis (Jeffreys, 1847)

Axinulus exintermedius (Gaglini, 1992)

Genaxinus eumyarius (M. Sars, 1870)

Leptaxinus incrassatus (Jeffreys, 1876)

Mendicula ferruginosa (Forbes, 1844)

Mendicula oblonga (Monterosato, 1880)

Parathyasira granulosa (Monterosato, 1874)

Thyasira biplicata (R. A. Philippi, 1836)

Thyasira subovata (Jeffreys, 1881)

Thyasira succisa (Jeffreys, 1876)

Order **Myida**

Family **Corbulidae**

Lentidium mediterraneum (O. G. Costa, 1830)

Varicorbula gibba (Olivi, 1792)

Family **Dreissenidae**

Mytilopsis leucophaeata (Conrad, 1831)

Family **Myidae**

Mya arenaria Linnaeus, 1758

Sphenia binghami W. Turton, 1822

Family **Pholadidae**

Barnea candida (Linnaeus, 1758)

Pholas dactylus Linnaeus, 1758

Family **Teredinidae**

Bankia carinata (J. E. Gray, 1827)

Lyrodus pedicellatus (Quatrefages, 1849)

Nototeredo norvagica (Spengler, 1792)

Psiloteredo megotara (Hanley, 1848)

Teredo navalis Linnaeus, 1758

Family **Xylophagaidae**

Xylophaga dorsalis (W. Turton, 1819)

Order **Venerida**

Family **Chamidae**

Chama circinata Monterosato, 1878

Chama gryphoides Linnaeus, 1758

Pseudochama gryphina (Lamarck, 1819)

Family **Glossidae**

Glossus humanus (Linnaeus, 1758)

Family **Kelliellidae**

Kelliella miliaris (R. A. Philippi, 1844)

Family **Mactridae**

Eastonia rugosa (Helbling, 1779)

Lutraria angustior R. A. Philippi, 1844

Lutraria lutraria (Linnaeus, 1758)

Lutraria oblonga (Gmelin, 1791)

Mactra glauca Born, 1778

Mactra stultorum (Linnaeus, 1758)

Spisula subtruncata (da Costa, 1778)

Family **Mesodesmatidae**

Donacilla cornea (Poli, 1791)

Family **Neoleptonidae**

Neolepton discriminatum Palazzi & Villari, 2001

Neolepton sulcatulum (Jeffreys, 1859)

Family **Trapezidae**

Coralliophaga lithophagella (Lamarck, 1819)

Family **Ungulinidae**

Diplodonta brocchii (Deshayes, 1850)

Diplodonta rotundata (Montagu, 1803)

Microstagon trigonum (Scacchi, 1835)

Family **Veneridae**

Callista chione (Linnaeus, 1758)

Chamelea gallina (Linnaeus, 1758)

Clausinella fasciata (da Costa, 1778)

Dosinia exoleta (Linnaeus, 1758)

Dosinia lupinus (Linnaeus, 1758)

Globivenus effossa (R. A. Philippi, 1836)

Gouldia minima (Montagu, 1803)

Irus irus (Linnaeus, 1758)

Lajonkairia lajonkairii (Payraudeau, 1826)

Mercenaria mercenaria (Linnaeus, 1758)

Mysia undata (Pennant, 1777)

Petricola lithophaga (Retzius, 1788)

Pitar mediterraneus (Aradas & Benoit, 1872)

Pitar rudis (Poli, 1795)

Polititapes aureus (Gmelin, 1791)

Polititapes lucens (Locard, 1886)

Polititapes rhomboides (Pennant, 1777)

Ruditapes decussatus (Linnaeus, 1758)

Ruditapes philippinarum (A. Adams & Reeve, 1850)

Timoclea ovata (Pennant, 1777)

Venerupis corrugata (Gmelin, 1791)

Venerupis geographica (Gmelin, 1791)

Venus casina Linnaeus, 1758

Venus nux Gmelin, 1791

Venus verrucosa Linnaeus, 1758

Subclass **Autobranchia**

Order **Carditida**

Family **Astartidae**

Astarte sulcata (da Costa, 1778)

Digitaria digitaria (Linnaeus, 1758)

Gonilia calliglypta (Dall, 1903)

Goodallia micalii Giribet & Peñas, 1999

Goodallia pusilla (Forbes, 1844)

Goodallia triangularis (Montagu, 1803)

Laevastarte fusca (Poli, 1795)

Family **Carditidae**

Cardita calyculata (Linnaeus, 1758)

Cardites antiquatus (Linnaeus, 1758)

Centrocardita aculeata (Poli, 1795)

Coripia corbis (R. A. Philippi, 1836)

Coripia jozinae (van Aartsen, 1985)

Glans trapezia (Linnaeus, 1767)

Family **Crassatellidae**

Crassatina modesta (H. Adams, 1869)

Superorder **Anomalodesmata**

Family **Clavagellidae**

Bryopa aperta (G. B. Sowerby I, 1823)

Bryopa melitensis (Broderip, 1834)

Family **Cuspidariidae**

Cardiomya costellata (Deshayes, 1835)

Cuspidaria cuspidata (Olivi, 1792)

Cuspidaria elliptica Di Geronimo, 1974

Cuspidaria rostrata (Spengler, 1793)

Tropidomya abbreviata (Forbes, 1843)

Family **Halonymphidae**

Halonympha depressa (Jeffreys, 1882)

Family **Lyonsiellidae**

Allogramma formosa (Jeffreys, 1882)

Policordia gemma (A. E. Verrill, 1880)

Family **Lyonsiidae**

Lyonsia norwegica (Gmelin, 1791)

Family **Pandoridae**

Pandora inaequivalvis (Linnaeus, 1758)

Pandora pinna (Montagu, 1803)

Family **Parilimyidae**

Parilimya loveni (Jeffreys, 1882)

Family **Periplomatidae**

Cochlodesma praetenue (Pulteney, 1799)

Family **Poromyidae**

Cetomya neaeroides (G. Seguenza, 1877)

Poromya granulata (Nyst & Westendorp, 1839)

Family **Thraciidae**

Thracia convexa (W. Wood, 1815)

Thracia corbuloides Deshayes, 1824

Thracia distorta (Montagu, 1803)

Thracia gracilis Jeffreys, 1865

Thracia phaseolina (Lamarck, 1818)

Thracia pubescens (Pulteney, 1799)

Thracia villosiuscula (MacGillivray, 1827)

Family **Verticordiidae**

Haliris granulata (G. Seguenza, 1860)

Haliris trapezoidea (G. Seguenza, 1876)

Class **Scaphopoda**

Order **Dentaliida**

Family **Dentaliidae**

Antalis agilis (M. Sars, 1872)

Antalis dentalis (Linnaeus, 1758)

Antalis inaequicostata (Dautzenberg, 1891)

Antalis panorma (Chenu, 1843)

Antalis vulgaris (da Costa, 1778)

Family **Fustiariidae**

Fustiaria rubescens (Deshayes, 1826)

Family **Gadilinidae**

Episiphon filum (G. B. Sowerby II, 1860)

Order **Gadilida**

Family **Entalinidae**

Entalina tetragona (Brocchi, 1814)

Family **Gadilidae**

Cadulus jeffreysii (Monterosato, 1875)

Cadulus subfusiformis (M. Sars, 1865)

Dischides politus (S. V. Wood, 1842)

Family **Pulsellidae**

Pulsellum lofotense (M. Sars, 1865)

Class **Gastropoda**

Subclass **Patellogastropoda**

Family **Lepetidae**

Iothia fulva (O. F. Müller, 1776)

Propilidium exiguum (W. Thompson, 1844)

Family **Lottiidae**

Lottia iani Scuderi, T. Nakano & Eernisse, 2021

Tectura virginea (O. F. Müller, 1776)

Family **Patellidae**

Patella caerulea Linnaeus, 1758

Patella ferruginea Gmelin, 1791

Patella rustica Linnaeus, 1758

Patella ulyssiponensis Gmelin, 1791

Subclass **Vetigastropoda**

Order **Lepetellida**

Family **Addisoniidae**

Addisonia excentrica (Tiberi, 1855)

Family **Anatomidae**

Anatoma aspera (R. A. Philippi, 1844)

Anatoma micalii D. L. Geiger, 2012

Anatoma tenuisculpta (G. Seguenza, 1880)

Anatoma umbilicata (Jeffreys, 1883)

Family **Fissurellidae**

Diodora dorsata (Monterosato, 1878)

Diodora gibberula (Lamarck, 1822)

Diodora graeca (Linnaeus, 1758)

Diodora italica (Defrance, 1820)

Emarginula adriatica O. G. Costa, 1830

Emarginula bonfittoi Smriglio & Mariottini, 2001

Emarginula christiaensi Piani, 1985

Emarginula fissura (Linnaeus, 1758)

Emarginula huzardii Payraudeau, 1826

Emarginula lorenzoi Fr. Giusti & Micali, 2019

Emarginula multistriata Jeffreys, 1882

Emarginula octaviana Coen, 1939

Emarginula punctulum Piani, 1980

Emarginula pustula Thiele, 1913

Emarginula rosea Bell, 1824

Emarginula sicula J. E. Gray, 1825

Emarginula solidula O. G. Costa, 1829

Emarginula tenera Locard, 1891

Emarginula tuberculosa Libassi, 1859

Fissurella nubecula (Linnaeus, 1758)

Fissurisepta granulosa Jeffreys, 1883

Puncturella noachina (Linnaeus, 1771)

Family **Haliotidae**

Haliotis stomatiaeformis Reeve, 1846

Haliotis tuberculata Linnaeus, 1758

Family **Larocheidae**

Trogloconcha yoidanyi Fr. Giusti, Pagli & Micali, 2018

Family **Lepetellidae**

Bogia labronica (Bogi, 1984)

Lepetella espinosae Dantart & Luque, 1994

Lepetella ionica F. Nordsieck, 1973

Family **Pseudococculinidae**

Copulabyssia tenuis (Monterosato, 1880)

Family **Scissurellidae**

Scissurella azorensis Nolt, 2008

Scissurella costata A. d'Orbigny, 1824

Sinezona cingulata (O. G. Costa, 1861)

Sinezona semicostata Burnay & Rolán, 1990

Order **Seguenziida**

Family **Chilodontaidae**

Danilia costellata (O. G. Costa, 1861)

Danilia tinei (Calcara, 1839)

Putzeysia wiseri (Calcara, 1842)

Vetulonia giacobbei Renda & Micali, 2016

Family **Pendromidae**

Rugulina fragilis (G. O. Sars, 1878)

Rugulina monterosatoi (van Aartsen & Bogi, 1987)

Family **Seguenzioidea *incertae sedis***

Adeuomphalus ammoniformis G. Seguenza, 1876

Adeuomphalus densicostatus (Jeffreys, 1884)

Akritogyra conspicua (Monterosato, 1880)

Anekes sculpturata Warén, 1992

Lissotesta gittenbergeri (van Aartsen & Bogi, 1988)

Lissotesta turrita (Gaglini, 1987)

Moelleriopsis messanensis (G. Seguenza, 1876)

Family **Trochaclididae**

Trochaclis versiliensis Warén, Carrozza & Rocchini, 1992

Order **Trochida**

Family **Calliostomatidae**

Calliostoma agrigentinum Coen, 1936

Calliostoma conulus (Linnaeus, 1758)

Calliostoma granulatum (Born, 1778)

Calliostoma laugieri (Payraudeau, 1826)

Calliostoma zizyphinum (Linnaeus, 1758)

Family **Colloniidae**

Cantrainea peloritana (Cantraine, 1835)

Homalopoma sanguineum (Linnaeus, 1758)

Family **Phasianellidae**

Tricolia deschampsi Gofas, 1993

Tricolia landinii Bogi & Campani, 2007

Tricolia miniata (Monterosato, 1884)

Tricolia pullus (Linnaeus, 1758)

Tricolia punctura Gofas, 1993

Tricolia speciosa (Megerle von Mühlfeld, 1824)

Tricolia tenuis (Michaud, 1829)

Family **Skeneidae**

Cirsonella romettensis (Granata Grillo, 1877)

Dasyskenea digeronimoi (La Perna, 1998)

Dikoleps depressa (Monterosato, 1880)

Dikoleps marianae Rubio, Dantart & Luque, 1998

Dikoleps pruinosa (Chaster, 1896)

Dikoleps templadoi Rubio, Dantart & Luque, 2004

Dikoleps umbilicostriata (Gaglini, 1987)

Lissomphalia bithynoides (Monterosato, 1880)

*Parviturbo laevisculptu*s Renda, Raveggi, Bartolini, Micali & Giacobbe, 2019

*Pseudorbis granulu*m (Brugnone, 1873)

Skenea basistriata (Jeffreys, 1877)

Skenea catenoides (Monterosato, 1877)

Skenea costulata Sbrana & Siracusa, 2018

Skenea divae Carrozza & van Aartsen, 2001

Skenea giemellorum Romani, Bogi & Bartolini, 2015

Skenea pelagia Nofroni & Valenti, 1987

Skenea serpuloides (Montagu, 1808)

Skeneoides exilissima (R. A. Philippi, 1844)

Skeneoides formosissima (Brugnone, 1873)

Family **Trochidae**

Callumbonella suturalis (R. A. Philippi, 1836)

Clanculus corallinus (Gmelin, 1791)

Clanculus cruciatus (Linnaeus, 1758)

Clanculus jussieui (Payraudeau, 1826)

Clelandella miliaris (Brocchi, 1814)

Gibbula ardens (Salis Marschlins, 1793)

Gibbula drepanensis (Brugnone, 1873)

Gibbula fanulum (Gmelin, 1791)

Gibbula guttadauri (R. A. Philippi, 1836)

Gibbula magus (Linnaeus, 1758)

Gibbula philberti (Récluz, 1843)

Gibbula tantilla Monterosato, 1890

Gibbula turbinoides (Deshayes, 1835)

Gibbula vimontiae Monterosato, 1884

Jujubinus baudoni (Monterosato, 1891)

Jujubinus curinii Bogi & Campani, 2006

Jujubinus errinae Smriglio, Mariottini & Giacobbe, 2016

Jujubinus exasperatus (Pennant, 1777)

Jujubinus fraterculus (Monterosato, 1880)

Jujubinus gravinae (Dautzenberg, 1881)

Jujubinus montagui (W. Wood, 1828)

Jujubinus seguenzae Ghisotti & Melone, 1975

Jujubinus striatus (Linnaeus, 1758)

Jujubinus tumidulus (Aradas, 1846)

Phorcus articulatus (Lamarck, 1822)

Phorcus mutabilis (R. A. Philippi, 1851)

Phorcus richardi (Payraudeau, 1826)

Phorcus turbinatus (Born, 1778)

Steromphala adansonii (Payraudeau, 1826)

Steromphala adriatica (R. A. Philippi, 1844)

Steromphala albida (Gmelin, 1791)

Steromphala divaricata (Linnaeus, 1758)

Steromphala leucophaea (R. A. Philippi, 1836)

Steromphala nebulosa (R. A. Philippi, 1849)

Steromphala racketti (Payraudeau, 1826)

Steromphala rarilineata (Michaud, 1829)

Steromphala umbilicaris (Linnaeus, 1758)

Steromphala varia (Linnaeus, 1758)

Family **Turbinidae**

Bolma rugosa (Linnaeus, 1767)

Subclass **Neomphaliones**

Order **Cocculinida**

Family **Bathysciadiidae**

Bathysciadium xylophagum Warén & Carrozza, 1995

Family **Cocculinidae**

Coccopigya spinigera (Jeffreys, 1883)

Coccopigya viminensis (Rocchini, 1990)

Subclass **Neritimorpha**

Order **Cycloneritida**

Family **Neritidae**

Smaragdia viridis (Linnaeus, 1758)

Subclass **Caenogastropoda**

Order **Littorinimorpha**

Family **Anabathridae**

Nodulus contortus (Jeffreys, 1856)

Nodulus spiralis van der Linden, 1986

Pisinna glabrata (Megerle von Mühlfeld, 1824)

Family **Aporrhaidae**

Aporrhais pespelecani (Linnaeus, 1758)

Aporrhais serresiana (Michaud, 1828)

Family **Assimineidae**

Assiminea gittenbergeri van Aartsen, 2008

Paludinella globularis (Hanley, 1844)

Paludinella sicana (Brugnone, 1876)

Family **Atlantidae**

Atlanta brunnea J. E. Gray, 1850

Atlanta helicinoidea J. E. Gray, 1850

Atlanta lesueurii J. E. Gray, 1850

Atlanta peronii Lesueur, 1817

Atlanta selvagensis de Vera & Seapy, 2006

Oxygyrus inflatus W. H. Benson, 1835

Protatlanta souleyeti (E. A. Smith, 1888)

Family **Barleeiidae**

Barleeia gougeti (Michaud, 1830)

Barleeia unifasciata (Montagu, 1803)

Family **Bursidae**

Talisman scrobilator (Linnaeus, 1758)

Family **Caecidae**

Caecum armoricum de Folin, 1869

Caecum auriculatum de Folin, 1868

Caecum clarkii P. P. Carpenter, 1859

Caecum subannulatum de Folin, 1870

Caecum trachea (Montagu, 1803)

Parastrophia asturiana de Folin, 1870

Family **Calyptraeidae**

Calyptraea chinensis (Linnaeus, 1758)

Crepidula fornicata (Linnaeus, 1758)

Crepidula moulinsii Michaud, 1829

Crepidula unguiformis Lamarck, 1822

Family **Capulidae**

Capulus ungaricus (Linnaeus, 1758)

Family **Carinariidae**

Carinaria lamarckii Blainville, 1817

Family **Cassidae**

Galeodea echinophora (Linnaeus, 1758)

Galeodea rugosa (Linnaeus, 1771)

Semicassis granulata (Born, 1778)

Semicassis saburon (Bruguière, 1792)

Semicassis undulata (Gmelin, 1791)

Family **Cerithiidae**

Bittium lacteum (R. A. Philippi, 1836)

Bittium latreillii (Payraudeau, 1826)

Bittium reticulatum (da Costa, 1778)

Cerithidium submammillatum (de Rayneval & Ponzi, 1854)

Cerithium alucastrum (Brocchi, 1814)

Cerithium lividulum Risso, 1826

Cerithium protractum Bivona, 1838

Cerithium renovatum Monterosato, 1884

Cerithium repandum Monterosato, 1878

Cerithium scabridum R. A. Philippi, 1848

Cerithium vulgatum Bruguière, 1792

Family **Cerithiopsidae**

Cerithiopsis atalaya R. B. Watson, 1885

Cerithiopsis carlottae Cecalupo & Perugia, 2018

Cerithiopsis diadema Monterosato, 1874

Cerithiopsis fayalensis R. B. Watson, 1880

Cerithiopsis iudithae Reitano & Buzzurro, 2006

Cerithiopsis jeffreysi R. B. Watson, 1885

Cerithiopsis ladae Prkic & Buzzurro, 2007

Cerithiopsis micalii (Cecalupo & Villari, 1997)

Cerithiopsis minima (Brusina, 1865)

Cerithiopsis nofronii Amati, 1987

Cerithiopsis perlata Monterosato, 1889

Cerithiopsis petanii Prkic & Mariottini, 2010

Cerithiopsis pulchresculpta Cachia, Mifsud & Sammut, 2004

Cerithiopsis scalaris Locard, 1891

Cerithiopsis tarruellasi Peñas & Rolán, 2006

Cerithiopsis tubercularis (Montagu, 1803)

Costulopsis buzzurroi (Cecalupo & Robba, 2010)

Costulopsis denticulata (Cecalupo & Robba, 2010)

Costulopsis nana (Jeffreys, 1867)

Dizoniopsis concatenata (Conti, 1864)

Dizoniopsis coppolae (Aradas, 1870)

Dizoniopsis zannii Cecalupo & Perugia, 2018

Krachia cylindrata (Jeffreys, 1885)

Krachia tiara (Monterosato, 1874)

Krachiopsis giannuzzii Smriglio & Mariottini, 1999

Seila trilineata (R. A. Philippi, 1836)

Family **Charoniidae**

Charonia lampas (Linnaeus, 1758)

Charonia seguenzae (Aradas & Benoit, 1871)

Family **Cingulopsidae**

Eatonina cossurae (Calcara, 1841)

Eatonina fulgida (J. Adams, 1797)

Eatonina ochroleuca (Brusina, 1869)

Eatonina pumila (Monterosato, 1884)

Tubbreva micrometrica (Aradas & Benoit, 1876)

Family **Cymatiidae**

Cabestana cutacea (Linnaeus, 1767)

Monoplex corrugatus (Lamarck, 1816)

Monoplex parthenopeus (Salis Marschlins, 1793)

Family **Cypraeidae**

Luria lurida (Linnaeus, 1758)

Naria spurca (Linnaeus, 1758)

Schilderina achatidea (Gray, 1837)

Zonaria pyrum (Gmelin, 1791)

Family **Elachisinidae**

Laeviphitus verduini van Aartsen, Bogi & Fr. Giusti, 1989

Family **Epitoniidae**

Acirsa subdecussata (Cantraine, 1835)

Cirsotrema pumiceum (Brocchi, 1814)

Epidendrium dendrophylliae (Bouchet & Warén, 1986)

Epitonium algerianum (Weinkauff, 1866)

Epitonium celesti (Aradas, 1854)

Epitonium clathrus (Linnaeus, 1758)

Epitonium finitimum (Monterosato, 1890)

Epitonium hispidulum (Monterosato, 1874)

Epitonium linctum (de Boury & Monterosato, 1890)

Epitonium muricatum (Risso, 1826)

Epitonium pulchellum (Bivona e Barnardi, 1832)

Epitonium spirilla (Monterosato, 1890)

Epitonium striatissimum (Monterosato, 1878)

Epitonium tiberii (de Boury, 1890)

Epitonium tryoni (de Boury, 1913)

Epitonium turtonis (W. Turton, 1819)

Gyroscala commutata (Monterosato, 1877)

Iphitus tenuisculptus (G. Seguenza, 1876)

Janthina exigua Lamarck, 1816

Janthina globosa Swainson, 1822

Janthina janthina (Linnaeus, 1758)

Janthina pallida W. Thompson, 1840

Narrimania concinna (Sykes, 1925)

Opalia abbotti Clench & R. D. Turner, 1952

Opalia coronata (R. A. Philippi & Scacchi, 1840)

Opalia crenata (Linnaeus, 1758)

Opaliopsis atlantis (Clench & R. D. Turner, 1952)

Papuliscala cerithielloides Bouchet & Warén, 1986

Punctiscala cerigottana (Sturany, 1896)

Family **Eratoidae**

Erato voluta (Montagu, 1803)

Family **Eulimidae**

Aclis ascaris (W. Turton, 1819)

Aclis attenuans Jeffreys, 1883

Aclis gulsonae (W. Clark, 1850)

Aclis trilineata R. B. Watson, 1897

Alcis minor (T. Brown, 1827)

Chileutomia miranda (Dautzenberg, 1925)

Crinophtheiros comatulicola (Graff, 1875)

Crinophtheiros giustii Gaglini, 1991

Curveulima beneitoi Peñas & Rolán, 2006

Curveulima dautzenbergi (Pallary, 1900)

Curveulima devians (Monterosato, 1884)

Ersilia mediterranea (Monterosato, 1869)

Eulima bilineata Alder, 1848

Eulima glabra (da Costa, 1778)

Fusceulima lineata (Monterosato, 1869)

Fusceulima minuta (Jeffreys, 1884)

Haliella stenostoma (Jeffreys, 1858)

Melanella alba (da Costa, 1778)

Melanella boscii (Payraudeau, 1826)

Melanella compactilis (Locard, 1891)

Melanella frielei (H. K. Jordan, 1895)

Melanella lubrica (Monterosato, 1890)

Melanella monterosatoi (Monterosato, 1890)

Melanella petitiana (Brusina, 1869)

Melanella polita (Linnaeus, 1758)

Melanella translucens (Monterosato, 1890)

Nanobalcis nana (Monterosato, 1878)

Parvioris ibizenca (F. Nordsieck, 1968)

Pelseneeria minor Koehler & Vaney, 1908

Sabinella bonifaciae (F. Nordsieck, 1974)

Sticteulima jeffreysiana (Brusina, 1869)

Vitreolina antiflexa (Monterosato, 1884)

Vitreolina cionella (Monterosato, 1878)

Vitreolina curva (Monterosato, 1874)

Vitreolina incurva (Bucquoy, Dautzenberg & Dollfus, 1883)

Vitreolina perminima (Jeffreys, 1883)

Vitreolina philippi (de Rayneval & Ponzi, 1854)

Family **Hydrobiidae**

Ecrobia ventrosa (Montagu, 1803)

Hydrobia acuta (Draparnaud, 1805)

Family **Iravadiidae**

Ceratia proxima (Forbes & Hanley, 1850)

Hyala vitrea (Montagu, 1803)

Family **Littorinidae**

Echinolittorina punctata (Gmelin, 1791)

Littorina littorea (Linnaeus, 1758)

Littorina saxatilis (Olivi, 1792)

Melarhaphe neritoides (Linnaeus, 1758)

Family **Naticidae**

Cryptonatica operculata (Jeffreys, 1885)

Euspira catena (da Costa, 1778)

Euspira fusca (Blainville, 1825)

Euspira guilleminii (Payraudeau, 1826)

Euspira macilenta (R. A. Philippi, 1844)

Euspira nitida (Donovan, 1803)

Naticarius hebraeus (Martyn, 1786)

Naticarius stercusmuscarum (Gmelin, 1791)

Neverita josephinia Risso, 1826

Notocochlis dillwynii (Payraudeau, 1826)

Payraudeautia intricata (Donovan, 1804)

Tectonatica prietoi (Hidalgo, 1873)

Tectonatica rizzae (R. A. Philippi, 1844)

Tectonatica sagraiana (A. d'Orbigny, 1842)

Family **Newtoniellidae**

Retilaskeya horrida (Monterosato, 1874)

Family **Ovulidae**

Pseudosimnia adriatica (G. B. Sowerby I, 1828)

Pseudosimnia carnea (Poiret, 1789)

Pseudosimnia juanjosensii (Pérez & Gómez, 1987)

Simnia aperta (G. B. Sowerby II, 1849)

Simnia spelta (Linnaeus, 1758)

Family **Pediculariidae**

Pedicularia sicula Swainson, 1840

Family **Planaxidae**

Fossarus ambiguus (Linnaeus, 1758)

Family **Potamididae**

Pirenella conica (Blainville, 1829)

Family **Pterotracheidae**

Firoloida desmarestia Lesueur, 1817

Pterotrachea coronata Forsskål, 1775

Pterotrachea hippocampus R. A. Philippi, 1836

Family **Ranellidae**

Ranella olearium (Linnaeus, 1758)

Family **Rissoidae**

Alvania aeoliae Palazzi, 1988

Alvania aliceae Amati, 2014

Alvania amatii Oliverio, 1986

Alvania aspera (R. A. Philippi, 1844)

Alvania beanii (Hanley, 1844)

Alvania cancellata (da Costa, 1778)

Alvania carinata (da Costa, 1778)

Alvania cimex (Linnaeus, 1758)

Alvania cimicoides (Forbes, 1844)

Alvania clathrella L. Seguenza, 1903

Alvania claudioi Buzzurro & Landini, 2007

Alvania desabatae Amati & Smriglio, 2016

Alvania dianiensis Oliverio, 1988

Alvania dipacoi Fr. Giusti & Nofroni, 1989

Alvania discors (T. Brown, 1818)

Alvania disparilis Monterosato, 1890

Alvania electa (Monterosato, 1874)

Alvania elegantissima (Monterosato, 1875)

Alvania elisae Margelli, 2001

Alvania geryonia (Nardo, 1847)

Alvania hallgassi Amati & Oliverio, 1985

Alvania hirta (Monterosato, 1884)

Alvania hispidula (Monterosato, 1884)

Alvania lactea (Michaud, 1830)

Alvania lanciae (Calcara, 1845)

Alvania lineata Risso, 1826

Alvania litoralis (F. Nordsieck, 1972)

Alvania lucinae Oberling, 1970

Alvania mamillata Risso, 1826

Alvania maximilicutiani Scuderi, 2014

Alvania nestaresi Oliverio & Amati, 1990

Alvania pagodula (Bucquoy, Dautzenberg & Dollfus, 1884)

Alvania peloritana (Aradas & Benoit, 1874)

Alvania pizzinii Amati, Smriglio & Oliverio, 2020

Alvania punctura (Montagu, 1803)

Alvania rominae Amati, Trono & Oliverio, 2020

Alvania rudis (R. A. Philippi, 1844)

Alvania scabra (R. A. Philippi, 1844)

Alvania scuderii Villari, 2017

Alvania settepassii Amati & Nofroni, 1985

Alvania skylla Tisselli & Micali, 2023

Alvania spinosa (Monterosato, 1890)

Alvania subareolata Monterosato, 1869

Alvania subcrenulata (Bucquoy, Dautzenberg & Dollfus, 1884)

Alvania subsoluta (Aradas, 1847)

Alvania tenera (R. A. Philippi, 1844)

Alvania testae (Aradas & Maggiore, 1844)

Alvania tomentosa (Pallary, 1920)

Alvania unica Amati & Quaggiotto, 2019

Alvania villarii Micali, Tisselli & Giunchi, 2005

Alvania weinkauffi Weinkauff, 1868

Alvania zetlandica (Montagu, 1816)

Alvania zylensis Gofas & Warén, 1982

Benthonella tenella (Jeffreys, 1869)

Botryphallus epidauricus (Brusina, 1866)

Crisilla beniamina (Monterosato, 1884)

Crisilla galvagni (Aradas & Maggiore, 1844)

Crisilla maculata (Monterosato, 1869)

Crisilla marioni (Fasulo & Gaglini, 1987)

Crisilla semistriata (Montagu, 1808)

Crisilla simulans (Locard, 1886)

Manzonia crassa (Kanmacher, 1798)

Obtusella intersecta (S. V. Wood, 1857)

Obtusella macilenta (Monterosato, 1880)

Onoba dimassai Amati & Nofroni, 1991

Onoba gianninii (F. Nordsieck, 1974)

Peringiella denticulata Ponder, 1985

Peringiella eburnea (F. Nordsieck, 1968)

Peringiella elegans (Locard, 1891)

Pusillina inconspicua (Alder, 1844)

Pusillina lineolata (Michaud, 1830)

Pusillina marginata (Michaud, 1830)

Pusillina munda (Monterosato, 1884)

Pusillina philippi (Aradas & Maggiore, 1844)

Pusillina radiata (R. A. Philippi, 1836)

Rissoa auriformis Pallary, 1904

Rissoa auriscalpium (Linnaeus, 1758)

Rissoa guerinii Récluz, 1843

Rissoa italiensis Verduin, 1985

Rissoa lia (Monterosato, 1884)

Rissoa membranacea (J. Adams, 1800)

Rissoa monodonta R. A. Philippi, 1836

Rissoa multicincta Smriglio & Mariottini, 1995

Rissoa panhormensis Verduin, 1985

Rissoa paradoxa (Monterosato, 1884)

Rissoa parva (da Costa, 1778)

Rissoa rodhensis Verduin, 1985

Rissoa scurra (Monterosato, 1917)

Rissoa similis Scacchi, 1836

Rissoa variabilis (Megerle von Mühlfeld, 1824)

Rissoa ventricosa Desmarest, 1814

Rissoa violacea Desmarest, 1814

Setia amabilis (Locard, 1886)

Setia ambigua (Brugnone, 1873)

Setia antipolitana (van der Linden & W. M. Wagner, 1987)

Setia fusca (R. A. Philippi, 1841)

Setia homerica Romani & Scuderi, 2015

Setia limpida Monterosato, 1884

Setia scillae (Aradas & Benoit, 1876)

Setia sciutiana (Aradas & Benoit, 1874)

Setia turriculata Monterosato, 1884

Family **Rissoinidae**

Rissoina bruguieri (Payraudeau, 1826)

Family **Scaliolidae**

Finella pupoides A. Adams, 1860

Family **Siliquariidae**

Petalopoma elisabettae Schiaparelli, 2002

Tenagodus obtusus (Schumacher, 1817)

Family **Skeneopsidae**

Skeneopsis planorbis (O. Fabricius, 1780)

Family **Tonnidae**

Eudolium crosseanum (Monterosato, 1869)

Tonna galea (Linnaeus, 1758)

Family **Tornidae**

Tornus subcarinatus (Montagu, 1803)

Family **Triphoridae**

Cheirodonta pallescens (Jeffreys, 1867)

Ionthoglossa pseudocanarica (Bouchet, 1985)

Marshallora adversa (Montagu, 1803)

Metaxia metaxa (Delle Chiaje, 1828)

Monophorus alboranensis Rolán & Peñas, 2001

Monophorus erythrosoma (Bouchet & Guillemot, 1978)

Monophorus perversus (Linnaeus, 1758)

Monophorus thiriotae Bouchet, 1985

Obesula marisnostri Bouchet, 1985

Similiphora similior (Bouchet & Guillemot, 1978)

Similiphora triclotae Bouchet, 1997

Strobiligera brychia (Bouchet & Guillemot, 1978)

Family **Triviidae**

Trivia arctica (Pulteney, 1799)

Trivia candidula (Gaskoin, 1836)

Trivia mediterranea (Risso, 1826)

Trivia monacha (da Costa, 1778)

Trivia multilirata (G. B. Sowerby II, 1870)

Family **Truncatellidae**

Truncatella subcylindrica (Linnaeus, 1767)

Family **Turritellidae**

Turritella turbona Monterosato, 1877

Turritellinella tricarinata (Brocchi, 1814)

Family **Vanikoridae**

Megalomphalus azoneus (Brusina, 1865)

Megalomphalus depressus (G. Seguenza, 1876)

Megalomphalus petitianus (Tiberi, 1868)

Megalomphalus yoidanyi Fr. Giusti, Nappo & Pagli, 2018

Talassia dagueneti (de Folin, 1873)

Family **Velutinidae**

Marsenia perspicua (Linnaeus, 1758)

Family **Vermetidae**

Dendropoma cristatum (Biondi-Giunti, 1859)

Dendropoma gaederopi (Mörch, 1861)

Petaloconchus glomeratus (Linnaeus, 1758)

Thylacodes arenarius (Linnaeus, 1758)

Thylaeodus rugulosus (Monterosato, 1878)

Thylaeodus semisurrectus (Bivona e Bernardi, 1832)

Vermetus granulatus (Gravenhorst, 1831)

Vermetus triquetrus Bivona e Bernardi, 1832

Family **Vitrinellidae**

Circulus striatus (R. A. Philippi, 1836)

Family **Xenophoridae**

Xenophora mediterranea Tiberi, 1863

Order **Neogastropoda**

Family **Borsoniidae**

Drilliola emendata (Monterosato, 1872)

Drilliola loprestiana (Calcara, 1841)

Typhlomangelia nivalis (Lovén, 1846)

Family **Cancellariidae**

Bivetiella cancellata (Linnaeus, 1767)

Bivetiella similis (G. B. Sowerby I, 1833)

Tribia coronata (Scacchi, 1835)

Family **Chauvetiidae**

Chauvetia affinis (Monterosato, 1889)

Chauvetia brunnea (Donovan, 1804)

Chauvetia candidissima (R. A. Philippi, 1836)

Chauvetia giunchiorum Micali, 1999

Chauvetia lefebvrii (Maravigna, 1840)

Chauvetia lineolata (Tiberi, 1868)

Chauvetia mamillata (Risso, 1826)

Chauvetia procerula (Monterosato, 1889)

Chauvetia recondita (Brugnone, 1873)

Chauvetia retifera (Brugnone, 1880)

Chauvetia tenuisculpta (Dautzenberg, 1891)

Chauvetia turritellata (Deshayes, 1835)

Chauvetia ventrosa F. Nordsieck, 1976

Family **Clathurellidae**

Pleurotomoides gracilis (Montagu, 1803)

Family **Colubrariidae**

Cumia intertexta (Helbling, 1779)

Family **Columbellidae**

Amphissa acutecostata (R. A. Philippi, 1844)

Columbella rustica (Linnaeus, 1758)

Mitrella canariensis (A. d'Orbigny, 1840)

Mitrella coccinea (R. A. Philippi, 1836)

Mitrella gervillii (Payraudeau, 1826)

Mitrella minor (Scacchi, 1836)

Mitrella psilla (Duclos, 1846)

Mitrella scripta (Linnaeus, 1758)

Mitrella svelta Kobelt, 1889

Family **Conidae**

Conus vayssierei Pallary, 1906

Conus ventricosus Gmelin, 1791

Family **Costellariidae**

Pusia ebenus (Lamarck, 1811)

Pusia granum (Forbes, 1844)

Pusia savignyi (Payraudeau, 1826)

Pusia tricolor (Gmelin, 1791)

Family **Cystiscidae**

Gibberula caelata (Monterosato, 1877)

Gibberula cristinae Tisselli, Agamennone & Giunchi, 2009

Gibberula jansseni van Aartsen, Menkhorst & Gittenberger, 1984

Gibberula miliaria (Linnaeus, 1758)

Gibberula philippii (Monterosato, 1878)

Gibberula recondita Monterosato, 1884

Gibberula simonae Smriglio, 2003

Gibberula turgidula (Locard & Caziot, 1900)

Family **Drilliidae**

Crassopleura maravignae (Bivona, 1838)

Spirotropis confusa (G. Seguenza, 1880)

Family **Fasciolariidae**

Aptyxis syracusana (Linnaeus, 1758)

Pseudofusus alternatus (Buzzurro & Russo, 2007)

Pseudofusus buzzurroi (Prkić & Russo, 2008)

Pseudofusus clarae (Russo & Renda, 2013)

Pseudofusus corallinus (Russo & Germanà, 2014)

Pseudofusus dimassai (Buzzurro & Russo, 2007)

Pseudofusus fioritae (Russo & Pagli, 2019)

Pseudofusus insularis (Russo & Calascibetta, 2018)

Pseudofusus labronicus Monterosato, 1884

Pseudofusus parvulus Monterosato, 1884

Pseudofusus pulchellus (R. A. Philippi, 1840)

Pseudofusus raricostatus (Del Prete, 1883)

Pseudofusus rostratus (Olivi, 1792)

Pseudofusus ventimigliae (Russo & Renda, 2013)

Tarantinaea lignaria (Linnaeus, 1758)

Family **Fusiturridae**

Fusiturris similis (Bivona, 1838)

Fusiturris undatiruga (Bivona e Bernardi, 1838)

Family **Granulinidae**

Granulina boucheti Gofas, 1992

Granulina gofasi Smriglio & Mariottini, 1996

Granulina guttula La Perna, 1999

Granulina marginata (Bivona e Bernardi, 1832)

Granulina melitensis Smriglio, Mariottini & Rufini, 1998

Granulina minusculina (Locard, 1897)

Granulina occulta (Monterosato, 1869)

Granulinopsis zanclea (Bogi, Boyer, Renda & Giacobbe, 2016)

Family **Horaiclavidae**

Haedropleura secalina (R. A. Philippi, 1844)

Haedropleura septangularis (Montagu, 1803)

Family **Mangeliidae**

Bela decussata (Locard, 1891)

Bela fuscata (Deshayes, 1835)

Bela menkhorsti van Aartsen, 1988

Bela nebula (Montagu, 1803)

Bela plicatilis (Risso, 1826)

Bela zenetouae (van Aartsen, 1988)

Bela zonata (Locard, 1891)

Benthomangelia macra (R. B. Watson, 1881)

Kurtziella serga (Dall, 1881)

Lyromangelia taeniata (Deshayes, 1835)

Mangelia callosa (F. Nordsieck, 1977)

Mangelia multilineolata (Deshayes, 1835)

Mangelia paciniana (Calcara, 1839)

Mangelia pontica Milaschewitsch, 1908

Mangelia scabrida Monterosato, 1890

Mangelia striolata Risso, 1826

Mangelia striolatoides Sabelli & Spada, 2023

Mangelia tenuisculpta Spada, 2023

Mangelia thapsiae (Oberling, 1970)

Mangelia unifasciata (Deshayes, 1835)

Pseudomangelia sicula (Reeve, 1846)

Pseudomangelia vauquelini (Payraudeau, 1826)

Pyrgocythara stosiciana (Brusina, 1869)

Smithiella costulata (Risso, 1826)

Sorgenfreispira brachystoma (R. A. Philippi, 1844)

Vexiguraleus hispidulus (Bellardi, 1847)

Villiersiella attenuata (Montagu, 1803)

Villiersiella tenuicosta (Brugnone, 1862)

Family **Marginellidae**

Volvarina mitrella (Risso, 1826)

Family **Mitridae**

Episcomitra cornicula (Linnaeus, 1758)

Episcomitra zonata (Marryat, 1819)

Isara cornea (Lamarck, 1811)

Family **Mitromorphidae**

Mitromorpha alyssae Amati, Smriglio & Oliverio, 2015

Mitromorpha bogii Amati, Smriglio & Oliverio, 2015

Mitromorpha columbellaria (Scacchi, 1836)

Mitromorpha karpathensis (F. Nordsieck, 1969)

Mitromorpha nofronii Amati, Smriglio & Oliverio, 2015

Mitromorpha olivoidea (Cantraine, 1835)

Family **Muricidae**

Babelomurex benoiti (Tiberi, 1855)

Babelomurex cariniferus (G. B. Sowerby II, 1834)

Babelomurex sentix (F. M. Bayer, 1971)

Babelomurex tectumsinense (Deshayes, 1856)

Bolinus brandaris (Linnaeus, 1758)

Coralliophila brevis (Blainville, 1832)

Coralliophila guancha Smriglio, Mariottini & Engl, 2003

Coralliophila meyendorffii (Calcara, 1845)

Coralliophila panormitana (Monterosato, 1869)

Coralliophila richardi (P. Fischer, 1882)

Coralliophila sofiae (Aradas & Benoit, 1876)

Coralliophila trigoi Mariottini, Smriglio & Rolán, 2005

Dermomurex scalaroides (Blainville, 1829)

Gracilipurpura craticulata (Bucquoy & Dautzenberg, 1882)

Hexaplex trunculus (Linnaeus, 1758)

Hirtomurex squamosus (Bivona e Bernardi, 1838)

Murexsul aradasii (Monterosato, 1883)

Murexsul cevikeri (Houart, 2000)

Muricopsis cristata (Brocchi, 1814)

Ocenebra baetica (Reeve, 1845)

Ocenebra edwardsii (Payraudeau, 1826)

Ocenebra erinaceus (Linnaeus, 1758)

Ocenebra helleri (Brusina, 1865)

Ocenebra ingloria (Crosse, 1865)

Ocenebra nicolai (Monterosato, 1884)

Ocenebra paddeui (Bonomolo & Buzzurro, 2006)

Ocenebra vazzanai Crocetta, Houart & Bonomolo, 2020

Ocinebrina aciculata (Lamarck, 1822)

Ocinebrina corallina (Scacchi, 1836)

Ocinebrina reinai Bonomolo & Crocetta, 2012

*Orania fusul*us (Brocchi, 1814)

Pagodula echinata (Kiener, 1839)

*Rapana venos*a (Valenciennes, 1846)

Stramonita haemastoma (Linnaeus, 1767)

Trophonopsis barvicensis (G. Johnston, 1825)

Trophonopsis muricata (Montagu, 1803)

Trophonopsis sparacioi Smriglio, Mariottini & Di Giulio, 2015

Typhinellus labiatus (De Cristofori & Jan, 1832)

Family **Nassariidae**

Tritia corniculum (Olivi, 1792)

Tritia corrugata (Brocchi, 1814)

Tritia elongata (Bucquoy, Dautzenberg & Dollfus, 1882)

Tritia grana (Lamarck, 1822)

Tritia incrassata (Strøm, 1768)

Tritia lima (Dillwyn, 1817)

Tritia mutabilis (Linnaeus, 1758)

Tritia neritea (Linnaeus, 1758)

Tritia nitida (Jeffreys, 1867)

Tritia pellucida (Risso, 1826)

Tritia tinei (Maravigna, 1840)

Tritia turulosa (Risso, 1826)

Tritia unifasciata (Kiener, 1834)

Tritia varicosa (W. Turton, 1825)

Family **Pisaniidae**

Aplus assimilis (Reeve, 1846)

Aplus coccineus (Monterosato, 1884)

Aplus dorbignyi (Payraudeau, 1826)

Aplus scacchianus (R. A. Philippi, 1844)

Enginella leucozona (R. A. Philippi, 1844)

Pisania striata (Gmelin, 1791)

Family **Raphitomidae**

Clathromangelia granum (R. A. Philippi, 1844)

Cyrillia aequalis (Jeffreys, 1867)

Cyrillia ephesina (Pusateri, Giannuzzi-Savelli & Stahlschmidt, 2017)

Cyrillia linearis (Montagu, 1803)

Gymnobela abyssorum (Locard, 1897)

Leufroyia concinna (Scacchi, 1836)

Leufroyia erronea Monterosato, 1884

Leufroyia leufroyi (Michaud, 1828)

Leufroyia villaria (Pusateri & Giannuzzi-Savelli, 2008)

Pleurotomella eurybrocha (Dautzenberg & H. Fischer, 1896)

Pleurotomella gibbera Bouchet & Warén, 1980

Pleurotomella packardii A. E. Verrill, 1872

Raphitoma alleryana (Sulliotti, 1889)

Raphitoma alternans (Monterosato, 1884)

Raphitoma atropurpurea (Locard & Caziot, 1900)

Raphitoma bartolinorum Pusateri & Giannuzzi-Savelli, 2018

Raphitoma bicolor (Risso, 1826)

Raphitoma brunneofasciata Pusateri, Giannuzzi-Savelli & Oliverio, 2013

Raphitoma contigua (Monterosato, 1884)

Raphitoma corbis (Potiez & Michaud, 1838)

Raphitoma cordieri (Payraudeau, 1826)

Raphitoma densa (Monterosato, 1884)

Raphitoma digiulioi Pusateri & Giannuzzi-Savelli, 2017

Raphitoma echinata (Brocchi, 1814)

Raphitoma farolita F. Nordsieck, 1977

Raphitoma griseomaculata Pusateri & Giannuzzi-Savelli, 2018

Raphitoma hispidella Giannuzzi-Savelli & Pusateri, 2019

Raphitoma horrida (Monterosato, 1884)

Raphitoma kharybdis Pusateri & Giannuzzi-Savelli, 2018

Raphitoma laviae (R. A. Philippi, 1844)

Raphitoma lineolata (Bucquoy, Dautzenberg & Dollfus, 1883)

Raphitoma locardi Pusateri, Giannuzzi-Savelli & Oliverio, 2013

Raphitoma melitis Kontadakis & Mbazios, 2019

Raphitoma papillosa (Pallary, 1904)

Raphitoma philberti (Michaud, 1829)

Raphitoma pseudohystrix (Sykes, 1906)

Raphitoma pumila (Monterosato, 1890)

Raphitoma pupoides (Monterosato, 1884)

Raphitoma radula (Monterosato, 1884)

Raphitoma skylla Pusateri & Giannuzzi-Savelli, 2018

Raphitoma smriglioi Pusateri & Giannuzzi-Savelli, 2013

Raphitoma spadiana Pusateri & Giannuzzi-Savelli, 2012

Raphitoma syrtensis F. Nordsieck, 1977

Taranis laevisculpta Monterosato, 1880

Taranis moerchi (Malm, 1861)

Teretia teres (Reeve, 1844)

Family **Tudiclidae**

Euthria cornea (Linnaeus, 1758)

Subclass **Heterobranchia**

Infraclass **"Lower Heterobranchia"**

Family **Architectonicidae**

Basisulcata lepida (C. Bayer, 1942)

Discotectonica discus (R. A. Philippi, 1844)

Heliacus fallaciosus (Tiberi, 1872)

Philippia hybrida (Linnaeus, 1758)

Pseudomalaxis zanclaeus (R. A. Philippi, 1844)

Pseudotorinia architae (O. G. Costa, 1841)

Solatisonax alleryi (G. Seguenza, 1876)

Solatisonax hemisphaerica (G. Seguenza, 1876)

Spirolaxis centrifuga (Monterosato, 1890)

Spirolaxis clenchi Jaume & Borro, 1946

Family **Cimidae**

Cima apicisbelli Rolán, 2003

Cima cuticulata Warén, 1993

Cima cylindrica (Jeffreys, 1856)

Cima minima (Jeffreys, 1858)

Graphis albida (Kanmacher, 1798)

Graphis gracilis (Monterosato, 1874)

Family **Cornirostridae**

Tomura depressa (Granata Grillo, 1877)

Tomura rubiorolanorum Romani & Sbrana, 2016

Family **Hyalogyrinidae**

Hyalogyra zibrowii Warén, 1997

Hyalogyrina amphorae Warén, Carrozza & Rocchini, 1997

Xenoskenea pellucida (Monterosato, 1874)

Family **Mathildidae**

Mathilda bieleri Smriglio & Mariottini, 2007

Mathilda cochlaeformis Brugnone, 1873

Mathilda coronata Monterosato, 1875

Mathilda gemmulata O. Semper, 1865

Mathilda letei Prkic & Smriglio, 2007

Mathilda quadricarinata (Brocchi, 1814)

Mathilda retusa Brugnone, 1873

Tuba jeffreysi (Dall, 1889)

Family **Omalogyridae**

Ammonicera columbretensis J. D. Oliver & Rolán, 2015

Ammonicera fischeriana (Monterosato, 1869)

Ammonicera nodulosa J. D. Oliver & Rolán, 2015

Omalogyra atomus (R. A. Philippi, 1841)

Omalogyra simplex (O. G. Costa, 1861)

Retrotortina fuscata Chaster, 1896

Family **Orbitestellidae**

Lurifax vitreus Warén & Bouchet, 2001

Orbitestella dariae (Liuzzi & Zucchi Stolfa, 1979)

Family **Xylodisculidae**

Xylodiscula boucheti Warén, Carrozza & Rocchini, 1992

Xylodiscula lens Warén, 1992

Xylodiscula wareni Bogi & Bartolini, 2008

Infraclass **Euthyneura**

Family **Acteonidae**

Acteon monterosatoi Dautzenberg, 1889

Acteon tornatilis (Linnaeus, 1758)

Callostracon tyrrhenicum (Smriglio & Mariottini, 1996)

Crenilabium exile (Jeffreys, 1870)

Japonactaeon pusillus (Forbes, 1844)

Rapturella globulina (Forbes, 1844)

Family **Aegiridae**

Aegires palensis Ortea, Luque & Templado, 1990

Family **Aeolidiidae**

Aeolidiella alderi (Cocks, 1852)

Anteaeolidiella lurana (Ev. Marcus & Er. Marcus, 1967)

Berghia coerulescens (Laurillard, 1832)

Berghia verrucicornis (A. Costa, 1867)

Cerberilla bernadettae J. Tardy, 1965

Limenandra nodosa Haefelfinger & Stamm, 1958

Spurilla neapolitana (Delle Chiaje, 1841)

Family **Aglajidae**

Aglaja tricolorata Renier, 1807

Camachoaglaja africana (Pruvot-Fol, 1953)

Melanochlamys algirae (A. Adams, 1850)

Melanochlamys miqueli (Pelorce, Horst & Hoarau, 2013)

Philinissima denticulata (J. Adams, 1800)

Philinopsis depicta (Renier, 1807)

Spinoaglaja wildpretii (Ortea, Bacallado & Moro, 2003)

Family **Akeridae**

Akera bullata O. F. Müller, 1776

Family **Alacuppidae**

Roxania monterosatoi Dautzenberg & H. Fischer, 1896

Roxania utriculus (Brocchi, 1814)

Family **Aplysiidae**

Aplysia dactylomela Rang, 1828

Aplysia depilans Gmelin, 1791

Aplysia fasciata Poiret, 1789

Aplysia punctata (Cuvier, 1803)

Bursatella leachii Blainville, 1817

Notarchus punctatus R. A. Philippi, 1836

Petalifera petalifera (Rang, 1828)

Phyllaplysia lafonti (P. Fischer, 1870)

Syphonota geographica (A. Adams & Reeve, 1850)

Family **Arminidae**

Armina maculata Rafinesque, 1814

Armina tigrina Rafinesque, 1814

Dermatobranchus rubidus (A. Gould, 1852)

Family **Bullidae**

Bulla striata Bruguière, 1792

Family **Cadlinidae**

Aldisa banyulensis Pruvot-Fol, 1951

Family **Calmidae**

Calma glaucoides (Alder & Hancock, 1854)

Calma gobioophaga Calado & Urgorri, 2002

Family **Calycidorididae**

Diaphorodoris alba Portmann & Sandmeier, 1960

Diaphorodoris luteocincta (M. Sars, 1870)

Diaphorodoris papillata Portmann & Sandmeier, 1960

Family **Cavoliniidae**

Cavolinia gibboides Rampal, 2002

Cavolinia inflexa (Lesueur, 1813)

Cavolinia tridentata (Forsskål, 1775)

Diacria trispinosa (Blainville, 1821)

Family **Chromodorididae**

Felimare fontandraui (Pruvot-Fol, 1951)

Felimare gasconi (Ortea, 1996)

Felimare orsinii (Vérany, 1846)

Felimare picta (R. A. Philippi, 1836)

Felimare tricolor (Cantraine, 1835)

Felimare villafranca (Risso, 1818)

Felimida binza (Ev. Marcus & Er. Marcus, 1963)

Felimida elegantula (R. A. Philippi, 1844)

Felimida krohni (Vérany, 1846)

Felimida luteorosea (Rapp, 1827)

Felimida purpurea (Risso, 1831)

Family **Cliidae**

Clio cuspidata (Bosc, 1801)

Clio pyramidata Linnaeus, 1767

Family **Colpodaspididae**

Colpodaspis pusilla M. Sars, 1870

Family **Creseidae**

Creseis acicula (Rang, 1828)

Creseis conica Eschscholtz, 1829

Styliola subula (Quoy & Gaimard, 1827)

Family **Cylichnidae**

Cylichna cylindracea (Pennant, 1777)

Family **Cymbuliidae**

Cymbulia parvidentata Pelseneer, 1888

Cymbulia peronii Blainville, 1818

Gleba cordata Forsskål, 1776

Family **Coryphellidae**

Coryphella lineata (Lovén, 1846)

Family **Dendrodorididae**

Dendrodoris grandiflora (Rapp, 1827)

Dendrodoris limbata (Cuvier, 1804)

Doriopsilla areolata Bergh, 1880

Doriopsilla rarispinosa Pruvot-Fol, 1951

Family **Diaphanidae**

Diaphana cretica (Forbes, 1844)

Diaphana minuta T. Brown, 1827

Family **Discodorididae**

Baptodoris cinnabarina Bergh, 1884

Carminodoris boucheti Ortea, 1979

Discodoris stellifera (Vayssière, 1903)

Jorunna tomentosa (Cuvier, 1804)

Paradoris indecora (Bergh, 1881)

Peltodoris atromaculata Bergh, 1880

Platydoris argo (Linnaeus, 1767)

Taringa armata Swennen, 1961

Taringa tritorquis Ortea, Perez & Llera, 1982

Tayuva lilacina (A. Gould, 1852)

Family **Dorididae**

Doris bertheloti (A. d'Orbigny, 1839)

Doris ocelligera (Bergh, 1881)

Doris verrucosa Linnaeus, 1758

Family **Dotidae**

Doto acuta Schmekel & Kress, 1977

Doto caballa Ortea, Moro & Bacallado, 2010

Doto cervicenigra Ortea & Bouchet, 1989

Doto coronata (Gmelin, 1791)

Doto floridicola Simroth, 1888

Doto fragaria Ortea & Bouchet, 1989

Doto koenneckeri Lemche, 1976

Doto paulinae Trinchese, 1881

Doto pontica Swennen, 1961

Doto pygmaea Bergh, 1871

Doto rosea Trinchese, 1881

Family **Embletoniidae**

Embletonia pulchra (Alder & Hancock, 1844)

Family **Eubranchidae**

Amphorina andra Korshunova, Malmberg, Prkić, Petani, Fletcher, Lundin & Martynov, 2020

Amphorina farrani (Alder & Hancock, 1844)

Amphorina linensis (García-Gómez, Cervera & F. J. Garcia, 1990)

Eubranchus exiguus (Alder & Hancock, 1848)

Eubranchus tricolor Forbes, 1838

Family **Facelinidae**

Caloria elegans (Alder & Hancock, 1845)

Caloria quatrefagesi (Vayssière, 1888)

Cratena peregrina (Gmelin, 1791)

Dicata odhneri Schmekel, 1967

Facelina annulicornis (Chamisso & Eysenhardt, 1821)

Facelina dubia Pruvot-Fol, 1948

Facelina fusca Schmekel, 1966

Facelina rubrovittata (A. Costa, 1866)

Facelina vicina (Bergh, 1882)

Facelinopsis marioni (Vayssière, 1888)

Favorinus branchialis (Rathke, 1806)

Favorinus ghanensis Edmunds, 1968

Family **Fionidae**

Fiona pinnata (Eschscholtz, 1831)

Family **Flabellinidae**

Calmella cavolini (Vérany, 1846)

Calmella gaditana (Cervera, García-Gómez & F. J. García, 1987)

Edmundsella pedata (Montagu, 1816)

Flabellina affinis (Gmelin, 1791)

Paraflabellina gabinierei (Vicente, 1975)

Paraflabellina ischitana (Hirano & T. E. Thompson, 1990)

Family **Goniodorididae**

Bermudella polycerelloides Ortea & Bouchet, 1983

Cargoa problematica (Pola, Paz-Sedano, Macali, Minchin, Marchini, Vitale, Licchelli & Crocetta, 2019)

Okenia elegans (Leuckart, 1828)

Okenia mediterranea (Ihering, 1886)

Pelagella castanea (Alder & Hancock, 1845)

Trapania lineata Haefelfinger, 1960

Trapania maculata Haefelfinger, 1960

Family **Haminoeidae**

Haloa japonica (Pilsbry, 1895)

Haminoea exigua (Schaefer, 1992)

Haminoea fusari (L. A. Alvarez, F. J. García & Villani, 1993)

Haminoea hydatis (Linnaeus, 1758)

Haminoea navicula (da Costa, 1778)

Haminoea orbignyana (A. Férussac, 1822)

Haminoea orteai Talavera, Murillo & Templado, 1987

Lamprohaminoea ovalis (Pease, 1868)

Roxaniella jeffreysi (Weinkauff, 1866)

Weinkauffia macandrewii (E. A. Smith, 1872)

Weinkauffia turgidula (Forbes, 1844)

Family **Hancockiidae**

Hancockia uncinata (Hesse, 1872)

Family **Heliconoididae**

Heliconoides inflatus (A. d'Orbigny, 1835)

Family **Hermaeidae**

Aplysiopsis elegans Deshayes, 1853

Cyerce cristallina (Trinchese, 1881)

Cyerce graeca T. E. Thompson, 1988

Hermaea bifida (Montagu, 1816)

Hermaea paucicirra Pruvot-Fol, 1953

Hermaea variopicta (A. Costa, 1869)

Family **Heroidae**

Hero blanchardi Vayssière, 1888

Family **Hyalocylidae**

Hyalocylis striata (Rang, 1828)

Family **Janolidae**

Antiopella cristata (Delle Chiaje, 1841)

Family **Laonidae**

Laona condensa (van der Linden, 1995)

Laona pruinosa (W. Clark, 1827)

Laona quadrata (S. V. Wood, 1839)

Family **Limacinidae**

Limacina bulimoides (A. d'Orbigny, 1835)

Limacina trochiformis (A. d'Orbigny, 1835)

Family **Limapontiidae**

Calliopaea bellula A. d'Orbigny, 1837

Ercolania coerulea Trinchese, 1892

Ercolania viridis (A. Costa, 1866)

Limapontia capitata (O. F. Müller, 1774)

Placida cremoniana (Trinchese, 1892)

Placida dendritica (Alder & Hancock, 1843)

Family **Myrrhinidae**

Dondice trainitoi Furfaro & Mariottini, 2020

Nemesignis banyulensis (Portmann & Sandmeier, 1960)

Family **Notodiaphanidae**

Notodiaphana atlantica Ortea, Moro & Espinosa, 2013

Family **Onchidorididae**

Acanthodoris pilosa (Abildgaard, 1789)

Idaliadoris neapolitana (Delle Chiaje, 1841)

Idaliadoris perlucea (Ortea & Moro, 2014)

Family **Oxynoidae**

Lobiger serradifalci (Calcara, 1840)

Oxynoe olivacea Rafinesque, 1814

Family **Peraclidae**

Peracle diversa (Monterosato, 1875)

Peracle reticulata (A. d'Orbigny, 1835)

Family **Philinidae**

Hermania scabra (O. F. Müller, 1784)

Philine angulata Jeffreys, 1867

Philine catena (Montagu, 1803)

Philine intricata Monterosato, 1884

Philine iris Tringali, 2001

Philine monterosati Monterosato, 1874

Philine punctata (J. Adams, 1800)

Philine quadripartita Ascanius, 1772

Philine striatula Monterosato, 1874

Philine vestita (R. A. Philippi, 1840)

Family **Phyllidiidae**

Phyllidia flava Aradas, 1847

Family **Piseinotecidae**

Piseinotecus soussi Tamsouri, Carmona, Moukrim & Cervera, 2014

Family **Plakobranchidae**

Bosellia mimetica Trinchese, 1891

Elysia flava A. E. Verrill, 1901

Elysia gordanae T. E. Thompson & Jaklin, 1988

Elysia margaritae Fez, 1962

Elysia rubeni Martín-Hervás, Carmona, K. R. Jensen, Licchelli, Vitale & Cervera, 2020

Elysia timida (Risso, 1818)

Elysia viridis (Montagu, 1804)

Thuridilla hopei (Vérany, 1853)

Family **Platyhedylidae**

Platyhedyle denudata Salvini-Plawen, 1973

Family **Pleurobranchaeidae**

Pleurobranchaea meckeli (Blainville, 1825)

Family **Pleurobranchidae**

Berthella aurantiaca (Risso, 1818)

Berthella elongata (Cantraine, 1835)

Berthella ocellata (Delle Chiaje, 1830)

Berthella perforata (R. A. Philippi, 1844)

Berthella stellata (Risso, 1826)

Berthellina edwardsii (Vayssière, 1897)

Pleurehdera stellata (Risso, 1826)

Pleurobranchus membranaceus (Montagu, 1816)

Pleurobranchus testudinarius Cantraine, 1835

Family **Polyceridae**

Crimora papillata Alder & Hancock, 1862

Kaloplocamus ramosus (Cantraine, 1835)

Limacia inesae Toms, Pola, Von der Heyden & Gosliner, 2021

Palio dubia (M. Sars, 1829)

Polycera elegans (Bergh, 1894)

Polycera hedgpethi Er. Marcus, 1964

Polycera quadrilineata (O. F. Müller, 1776)

Polycerella emertoni A. E. Verrill, 1880

*Thecacera pennigera (*Montagu, 1813)

Family **Retusidae**

Pyrunculus hoernesi (Weinkauff, 1866)

Pyrunculus ovatus (Jeffreys, 1871)

Retusa crebrisculpta (Monterosato, 1884)

Retusa crossei (Bucquoy, Dautzenberg & Dollfus, 1886)

Retusa laevisculpta (Granata Grillo, 1877)

Retusa leptoeneilema (Brusina, 1866)

Retusa mammillata (R. A. Philippi, 1836)

Retusa minutissima (Monterosato, 1878)

Retusa nitidula (Lovén, 1846)

Retusa parvula (Jeffreys, 1883)

Retusa truncatula (Bruguière, 1792)

Retusa umbilicata (Montagu, 1803)

Family **Rhizoridae**

*Volvulella acumina*ta (Bruguière, 1792)

Family **Ringiculidae**

Ringicula auriculata (Ménard de la Groye, 1811)

Ringicula ciommeii Mariottini, Smriglio & Oliverio, 2000

Ringicula conformis Monterosato, 1877

Ringicula gianninii F. Nordsieck, 1974

Family **Rissoellidae**

Rissoella diaphana (Alder, 1848)

Rissoella inflata (Monterosato, 1880)

Rissoella opalina (Jeffreys, 1848)

Family **Runcinidae**

Runcina adriatica T. E. Thompson, 1980

Runcina brenkoae T. E. Thompson, 1980

Runcina lupiaensis A. K. Araujo, Pola, Malaquias, Vitale & Cervera, 2023

Runcina ornata (Quatrefages, 1844)

Family **Samlidae**

Luisella babai (Schmekel, 1972)

Family **Scaphandridae**

Scaphander lignarius (Linnaeus, 1758)

Family **Scyllaeidae**

Scyllaea pelagica Linnaeus, 1758

Family **Siphonariidae**

Williamia gussoni (O. G. Costa, 1829)

Family **Tethydidae**

Melibe viridis (Kelaart, 1858)

Tethys fimbria Linnaeus, 1767

Family **Trinchesiidae**

Catriona gymnota (Couthouy, 1838)

Catriona maua Ev. Marcus & Er. Marcus, 1960

Rubramoena amoena (Alder & Hancock, 1845)

Trinchesia caerulea (Montagu, 1804)

Trinchesia genovae (O'Donoghue, 1926)

Trinchesia miniostriata Schmekel, 1968

Trinchesia morrowae Korshunova, Picton, Furfaro, Mariottini, Pontes, Prkić, Fletcher, Malmberg, Lundin & Martynov, 2019

Trinchesia ocellata Schmekel, 1966

Family **Tritoniidae**

Candiella lineata (Alder & Hancock, 1848)

Candiella manicata (Deshayes, 1853)

Candiella odhneri (J. Tardy, 1963)

Candiella striata (Haefelfinger, 1963)

Marionia blainvillea (Risso, 1818)

Family **Tylodinidae**

Tylodina duebenii Lovén, 1846

Tylodina perversa (Gmelin, 1791)

Tylodina rafinesquii R. A. Philippi, 1836

Family **Umbraculidae**

Spiricella unguiculus Rang, 1828

Umbraculum umbraculum ([Lightfoot], 1786)

Family **Volvatellidae**

Ascobulla fragilis (Jeffreys, 1856)

Superorder **Pylopulmonata**

Family **Amathinidae**

Carinorbis clathrata (R. A. Philippi, 1844)

Family **Pyramidellidae**

Auristomia erjaveciana (Brusina, 1869)

Auristomia fusulus (Monterosato, 1878)

Auristomia ignorata Monterosato, 1917

Brachystomia angusta (Jeffreys, 1867)

Brachystomia eulimoides (Hanley, 1844)

Brachystomia pizzinii Nofroni, Renda & Vannozzi, 2022

Brachystomia scalaris (MacGillivray, 1843)

Careliopsis modesta (de Folin, 1870)

Doliella nitens (Jeffreys, 1870)

Eulimella acicula (R. A. Philippi, 1836)

Eulimella ataktos Warén, 1991

Eulimella bogii van Aartsen, 1994

Eulimella cerullii (Cossmann, 1915)

Eulimella cossignaniorum van Aartsen, 1994

Eulimella hoeisaeteri Micali, 2021

Eulimella neoattenuata Gaglini, 1992

Eulimella scillae (Scacchi, 1835)

Eulimella superflua (Monterosato, 1875)

Eulimella ventricosa (Forbes, 1844)

Euparthenia bulinea (R. T. Lowe, 1841)

Euparthenia humboldti (Risso, 1826)

Folinella excavata (R. A. Philippi, 1836)

Folinella ghisottii van Aartsen, 1984

Kejdonia cachiai (Mifsud, 1998)

Liostomia afzelii Warén, 1991

Liostomia clavula (Lovén, 1846)

Liostomia hansgei Warén, 1991

Liostomia mamoi Mifsud, 1993

*Megastomia alungata (*F. Nordsieck, 1972)

Megastomia conoidea (Brocchi, 1814)

Megastomia conspicua (Alder, 1850)

Noemiamea dolioliformis (Jeffreys, 1848)

Odostomella bicincta (Tiberi, 1868)

Odostomella doliolum (R. A. Philippi, 1844)

Odostomia aartseni Nofroni, 1988

Odostomia acuta Jeffreys, 1848

Odostomia improbabilis Oberling, 1970

Odostomia kromi van Aartsen, Menkhorst & Gittenberger, 1984

Odostomia lorellae Micali, 1987

Odostomia lukisii Jeffreys, 1859

Odostomia megerlei (Locard, 1886)

Odostomia nardoi Brusina, 1869

Odostomia plicata (Montagu, 1803)

Odostomia silesui Nofroni, 1988

Odostomia striolata Forbes & Hanley, 1850

Odostomia suboblonga Jeffreys, 1884

Odostomia turriculata Monterosato, 1869

Odostomia turrita Hanley, 1844

Odostomia unidentata (Montagu, 1803)

Ondina anceps Gaglini, 1992

Ondina crystallina Locard, 1891

Ondina diaphana (Jeffreys, 1848)

Ondina obliqua (Alder, 1844)

Ondina vitrea (Brusina, 1866)

Ondina warreni (W. Thompson, 1845)

Parthenina angulosa (Monterosato, 1889)

Parthenina brattstroemi (Warén, 1991)

Parthenina clathrata (Jeffreys, 1848)

Parthenina dantarti (Peñas & Rolán, 2008)

Parthenina decussata (Montagu, 1803)

Parthenina dollfusi Kobelt, 1903

Parthenina emaciata (Brusina, 1866)

Parthenina flexuosa (Monterosato, 1874)

Parthenina indistincta (Montagu, 1808)

Parthenina interstincta (J. Adams, 1797)

Parthenina juliae (de Folin, 1872)

Parthenina monozona (Brusina, 1869)

Parthenina monterosatii (Clessin, 1900)

Parthenina moolenbeeki (Amati, 1987)

Parthenina palazzii (Micali, 1984)

Parthenina penchynati (Bucquoy, Dautzenberg & Dollfus, 1883)

Parthenina rinaldii (Micali & Nofroni, 2004)

Parthenina suturalis (R. A. Philippi, 1844)

Parthenina terebellum (R. A. Philippi, 1844)

Pyrgiscus crenatus (T. Brown, 1827)

Pyrgiscus jeffreysii (Jeffreys, 1848)

Pyrgiscus rufus (R. A. Philippi, 1836)

Pyrgolidium internodulum (S. V. Wood, 1848)

Pyrgostylus striatulus (Linnaeus, 1758)

Pyrgulina stefanisi (Jeffreys, 1869)

Spiralina alpinoligustica (Sacco, 1892)

Strioturbonilla sigmoidea (Monterosato, 1880)

Syrnola minuta H. Adams, 1869

Tiberia minuscula (Monterosato, 1880)

Tibersyrnola unifasciata (Forbes, 1844)

Trabecula jeffreysiana Monterosato, 1884

Tragula fenestrata (Jeffreys, 1848)

Turbonilla acuta (Donovan, 1804)

Turbonilla acutissima Monterosato, 1884

Turbonilla amoena (Monterosato, 1878)

Turbonilla flaianoi Mazziotti, Agamennone, Micali & Tisselli, 2006

Turbonilla gradata Bucquoy, Dautzenberg & Dollfus, 1883

Turbonilla hamata F. Nordsieck, 1972

Turbonilla lactea (Linnaeus, 1758)

Turbonilla magnifica G. Seguenza, 1880

Turbonilla micans (Monterosato, 1875)

Turbonilla multilirata (Monterosato, 1875)

Turbonilla paucistriata (Jeffreys, 1884)

Turbonilla postacuticostata Sacco, 1892

Turbonilla pumila G. Seguenza, 1876

Turbonilla pusilla (R. A. Philippi, 1844)

Turbonilla rosewateri Corgan & van Aartsen, 1993

Turbonilla sinuosa (Jeffreys, 1884)

Superorder **Eupulmonata**

Family **Ellobiidae**

Leucophytia bidentata (Montagu, 1808)

Myosotella myosotis (Draparnaud, 1801)

Ovatella firminii (Payraudeau, 1826)

Family **Onchidiidae**

Onchidella celtica (Audouin & Milne-Edwards, 1832)

Family **Otinidae**

Otina ovata (T. Brown, 1827)

Family **Trimusculidae**

Trimusculus mammillaris (Linnaeus, 1758)

Infraclass **Mesoneura**

Family **Murchisonellidae**

Ebala gradata (Monterosato, 1878)

Ebala nitidissima (Montagu, 1803)

Ebala pointeli (de Folin, 1868)

Ebala striatula (Jeffreys, 1856)

Pseudochileutomia carinata (de Folin, 1870)

Family **Tjaernoeiidae**

Tjaernoeia exquisita (Jeffreys, 1883)

Tjaernoeia unisulcata (Chaster, 1897)
